# Synchronization within synchronization: transients and intermittency in ecological networks

**DOI:** 10.1093/nsr/nwaa269

**Published:** 2020-10-24

**Authors:** Huawei Fan, Ling-Wei Kong, Xingang Wang, Alan Hastings, Ying-Cheng Lai

**Affiliations:** School of Physics and Information Technology, Shaanxi Normal University, Xi’an 710062, China; School of Electrical, Computer and Energy Engineering, Arizona State University, Tempe, AZ 85287, USA; School of Electrical, Computer and Energy Engineering, Arizona State University, Tempe, AZ 85287, USA; School of Physics and Information Technology, Shaanxi Normal University, Xi’an 710062, China; Department of Environmental Science and Policy, University of California, Davis, CA 95616, USA; Santa Fe Institute, Santa Fe, NM 87501, USA; School of Electrical, Computer and Energy Engineering, Arizona State University, Tempe, AZ 85287, USA; Department of Physics, Arizona State University, Tempe, AZ 85287, USA

**Keywords:** ecological networks, cluster synchronization, phase synchronization, transient chaos, network symmetry

## Abstract

Transients are fundamental to ecological systems with significant implications to management, conservation and biological control. We uncover a type of transient synchronization behavior in spatial ecological networks whose local dynamics are of the chaotic, predator–prey type. In the parameter regime where there is phase synchronization among all the patches, complete synchronization (i.e. synchronization in both phase and amplitude) can arise in certain pairs of patches as determined by the network symmetry—henceforth the phenomenon of  ‘synchronization within synchronization.’ Distinct patterns of complete synchronization coexist but, due to intrinsic instability or noise, each pattern is a transient and there is random, intermittent switching among the patterns in the course of time evolution. The probability distribution of the transient time is found to follow an algebraic scaling law with a divergent average transient lifetime. Based on symmetry considerations, we develop a stability analysis to understand these phenomena. The general principle of symmetry can also be exploited to explain previously discovered, counterintuitive synchronization behaviors in ecological networks.

## Teaser

A surprising transient synchronization behavior in spatial ecological networks is uncovered: under global phase synchronization, a subset of nodes determined by symmetry can synchronize in amplitude in an intermittent fashion.

## INTRODUCTION

Synchronization in spatially extended ecological systems has been a topic of continuous interest [1–14]. In a variety of ecosystems, cyclic patterns across space that persist in time are ubiquitous, in which synchronous dynamics are believed to play an important role [2,3,11,14]. For example, in a network of predator–prey systems, chaotic phase synchronization was uncovered, providing an explanation for a class of ecological cycles, e.g. the hare-lynx cycle [15–19], in which the populations in different spatial regions oscillate synchronously and periodically in phase but their peak abundances are different and vary erratically with time [2,3]. More recently, synchronous dynamics were exploited to explain the correlations across space of cyclic dynamics in ecology, especially in terms of yield from pistachio trees [11,14]. Based on a large data set from over 6500 trees in a pistachio orchard in California, the authors established a surprising link between the spatially networked system of pistachio trees and the Ising model in statistical physics, with the common trait that local, neighbor-to-neighbor interactions (root grafting for the former and spin interactions for the latter) can generate correlation and synchronization over large distances.

In ecology, the importance of transient dynamics has been increasingly recognized [20–24], making uncovering and understanding ecological transients a frontier area of research [25]. In this paper, we report a class of transient synchronization behaviors in a spatially distributed ecological network of patches, each with a chaotic predator–prey type of dynamics. The oscillators are locally coupled and, for simplicity, they are located on a topological circle in space. Each oscillator describes the population dynamics of a patch, in which there are three interacting species: vegetation, herbivores and predators. When isolated, the dynamics of the oscillators are chaotic. In the presence of local coupling, chaotic phase synchronization prevails [2,3,26]. Our main finding is that, enclosed within phase synchronization, complete synchronization in both phase and amplitude of the abundance oscillations emerges among certain subsets of patches. The subsets are determined by the intrinsic symmetries of the network, i.e. each symmetry generates a specific configuration of the subsets (or clusters of oscillators). There is then cluster synchronization. As complete synchronization among a subset of patches occurs under the umbrella of phase synchronization among *all* the patches, we call this phenomenon ‘synchronization within synchronization.’ The striking behavior is that the synchronous dynamics associated with any configuration are *transient*: any cluster synchronization can be maintained for only a finite amount of time when the network is subject to intrinsic stochasticity (due to chaos) and/or random noise of arbitrarily small amplitude. When one form of cluster synchronization breaks down, a new form of cluster synchronization allowed by the system symmetry emerges. In the course of time evolution, there is intermittent switching among the distinct patterns of cluster synchronization. The duration of any cluster synchronization state, or the transient time, is found to obey an algebraic scaling law. Mathematically, the emergence of transient cluster synchronization, intermittency and the distribution of the transient lifetime can be understood through a dynamical stability analysis based on symmetry considerations. Ecologically, in addition to uncovering transients in patch synchronization dynamics, our finding implies that the ubiquitous phenomenon of population cycles can possess a more organized dynamical structure than previously thought: not only do the populations in all patches exhibit the same trend of variation (synchronized in phase), but certain patches can also have the same population at any time even if they are not directly coupled and are separated by a large distance. In fact, nearby patches, in spite of being directly coupled, may not be completely synchronized. The results establish the possibility and the dynamical mechanism for spatially ‘remote’ synchronization in ecological systems.

We remark that, in the field of complex dynamical systems, the phenomenon of cluster synchronization has been investigated [27–30]. For example, it was found earlier that long-range links added to a loop network can induce cluster synchronization patterns [27]. Removing links or adding weights to links can affect the stability of cluster synchronization and induce switching among different patterns of synchronization [28]. In a symmetric network of coupled identical phase oscillators, phase lags can induce cluster synchronization [29]. These previous studies established a fundamental connection between the symmetry of the network and the patterns of cluster synchronization, and a computational group theory was developed [30] to understand this connection. For example, a group can be generated by the possible symmetries of a network and the orbits of the symmetry group determine the partition of the synchronous clusters. In general, the phase space of the whole networked dynamical system can be decomposed into the synchronization subspace and the transverse subspace through a transformation matrix generated by the symmetry group, which determines the stability of the cluster synchronization patterns. In the existing literature on cluster synchronization, there are two common features: (1) the clusters are desynchronized from each other, in both phase and amplitude, and (2) a cluster synchronization state is sustained. In addition, the phenomenon of intermittent synchronization was studied, where the system switches between cluster and global synchronizations [31], a phenomenon that is usually induced by noise [32]. Quite distinctively, the transient cluster synchronization state uncovered in this paper has the following features. (1) The clusters are synchronized in phase, and (2) the emergence of the cluster configuration is time dependent and in fact transient: it can alter in an intermittent fashion where the system switches between different cluster synchronization states. To our knowledge, the phenomena uncovered in this paper, namely transient cluster synchronization umbrellaed by chaotic phase synchronization and intermittent switching among the coexisting cluster synchronization patterns, were not known previously. The phenomena enrich our knowledge about the interplay between network symmetry and the collective dynamics, and are broadly interesting to researchers from different fields including physics, complex systems and ecology.

## RESULTS

We consider the following vertical food web network model [2]:

(1a) \begin{eqnarray*} \dot{x}_{i} & =& a x_{i}-\alpha _1 f_1(x_{i},y_{i}), \end{eqnarray*}

(1b) \begin{eqnarray*} \dot{y}_{i} &=& -b y_{i}+\alpha _1 f_1(x_i,y_i)-\alpha _2 f_2(y_{i},z_{i})\nonumber\\ &&+\,\varepsilon _y\sum ^{N}_{j=1}a_{ij}(y_{j}-y_{i}), \end{eqnarray*}

(1c) \begin{eqnarray*} \dot{z}_{i} &=& -c(z_{i}-z_0)+\alpha _2 f_2(y_{i},z_{i})\nonumber\\ &&+\varepsilon _z\sum ^{N}_{j=1}a_{ij}(z_{j}-z_{i}). \end{eqnarray*}

**Figure 1.**
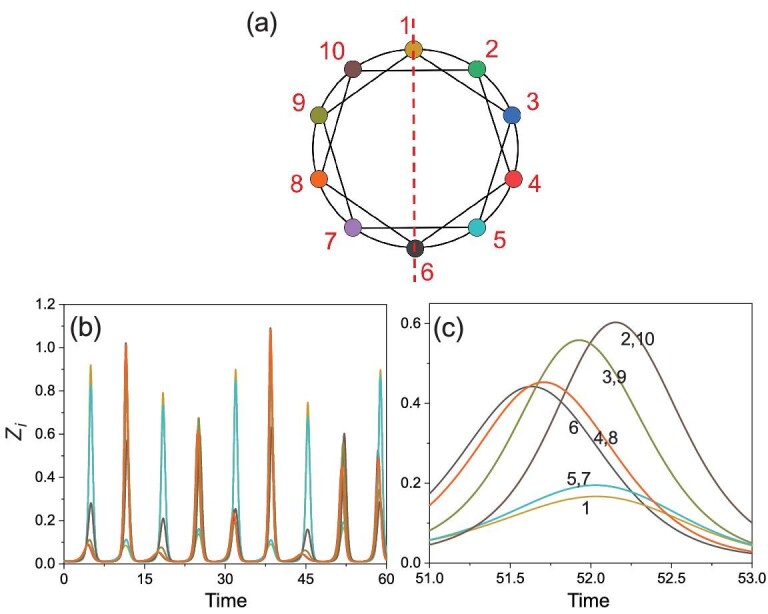
Network structure, chaotic phase synchronization, and evidence of cluster synchronization. (a) A dispersal network of ten patches with a regular ring structure. Each node has four links: two to the nearest neighbors and two to the next nearest neighbors. The red dashed line specifies the symmetry axis. (b) Representative time series of the ten predator populations $z$_*i*_ for ϵ = 0.038. The phases of the chaotic oscillators are synchronized, as the peaks of all predator populations are locked with each other. (c) A magnification of a single peak of the time series in (b), where there are six distinct time series, indicating that the four remaining time series coincide completely with some of the six distinct time series. In fact, there are four pairs of patches, (2,10), (3,9), (4,8) and (5,7), and both the amplitude and phase of the paired patches are synchronized—complete synchronization, signifying network cluster synchronization.

Here *i*, *j* = 1, …, *N* are the oscillator (patch) indices, and the dynamical variables *x*_*i*_, *y*_*i*_ and $z$_*i*_ represent the abundances of vegetation, herbivores and predators in patch *i*, respectively. The consumer–resource and predator–prey interactions are represented by the Holling type-II term *f*_1_(*x*, *y*) = *xy*/(1 + β*x*) and the Lotka-Volterra term *f*_2_(*y*, $z$) = *yz*, respectively. For the parameter setting (*a*, *b*, *c*, $z$_0_, α_1_, α_2_, β) = (1, 1, 10, 6 × 10^−3^, 0.2, 1, 5 × 10^−2^), the local dynamics of each patch display the feature of uniform phase growth and chaotic amplitude commonly observed in ecological and biological systems [33]. In fact, with this set of parameter values, the individual isolated nodal dynamics reproduce the time series of lynx abundances observed from six different regions in Canada during the period from 1821 to 1934 [2]. The patches are coupled through the migrations of herbivores (*y*) and predators ($z$), with the respective coupling parameters ϵ_*y*_ and ϵ_$z$_. The coupling relationship of the patches, namely the network structure, is described by the adjacency matrix *A* = {*a*_*ij*_}: *a*_*ij*_ = *a*_*ji*_ = 1 if patches *i* and *j* are connected; otherwise, *a*_*ij*_ = 0. Ecologically, food web networks usually are not large [2,34]. Following the setting in [34], we study a small regular ring network of *N* = 10 discrete habitat patches, as illustrated in Fig. 1(a). The phenomenon to be reported below also occurs for different parameter values, e.g. for 0.7 ≤ *b* ≤ 1.2.

### Emergence of cluster synchronization

We focus on the case in which ϵ_*y*_ = ϵ_$z$_ ≡ ϵ. (The general case of ϵ_*y*_ ≠ ϵ_$z$_ is treated in Section I of the online supplementary material [SM]) It was shown previously [2] that, while the species in different patches exhibit chaotic variations, phase synchronization among the populations in all patches can arise. That is, the populations exhibit exactly the same trend of ups and downs, giving rise to certain degree of spatial correlation or coherence. An example of chaotic phase synchronization is shown in Fig. 1(b), where the time series of the predator species $z$_*i*_ in all patches are displayed. It can be seen that the highs of the ten populations occur in the same time intervals, so are the lows. The amplitudes of the population variations are chaotic and apparently not synchronized. If there is an absolute lack of any synchronization in amplitude, the ten time series should all have been distinct. However, a careful examination of the time series reveals fewer than ten distinct traces; as shown in Fig. 1(c), there are only six distinct time series, among which the population amplitudes of the following four pairs of patches are completely synchronized: (5,7), (4,8), (3,9), (2,10) (patch 1 is not synchronized in amplitude with any other patch, neither is patch 6). The remarkable phenomenon is the emergence of complete synchronization in both phase and amplitude between patches that are not directly coupled with each other, such as patches 4 and 8 as well as 3 and 9. For any one of these four patches, its population chooses to synchronize not with that of the nearest neighbor or that of the second nearest neighbor (i.e. a directly coupled patch), but with that of a relatively remote one. That is, for the coupled chaotic food web network, while previous work [2,3] revealed that the populations of all spatial patches vary coherently in phase, a stronger level of coherence, i.e. synchronization in both phase and amplitude, can emerge spontaneously between spatially remote patches.

### Intermittency associated with cluster synchronization

**Figure 2.**
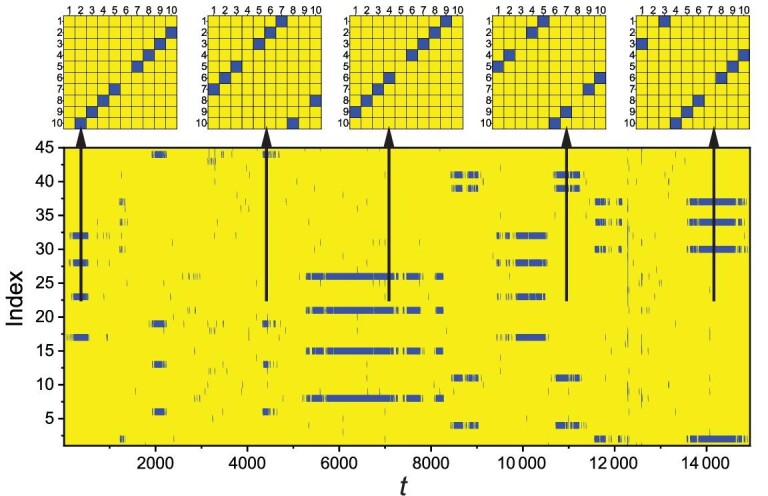
Cluster synchronization within chaotic phase synchronization and intermittent switching. Shown is the time evolution of the matrix elements *c*_*ij*_(*t*) for ϵ = 0.038, where the elements of one are marked blue and the others are marked yellow. In the top panel, there are five distinct matrices, indicating five cluster synchronization states or patterns. The corresponding time series are displayed in the bottom panel. The index marks the element position of the upper triangular part of *c*_*ij*_ and time *t* is rescaled by the average period of the population oscillations. Each vertical arrow indicates the time interval in which a specific cluster synchronization state appears.

To characterize cluster synchronization within chaotic phase synchronization, we define the following synchronization matrix ${\mathcal {C}}(t)$ with element *c*_*ij*_(*t*): *c*_*ij*_(*t*) = *c*_*ji*_(*t*) = 1 if the difference between the predator populations of patches *i* and *j* is sufficiently small, e.g. |$z$_*j*_(*t*) − $z$_*i*_(*t*)| < 10^−4^, and *c*_*ij*_(*t*) = 0 otherwise. As shown in the top row of Fig. 2, for ϵ = 0.038, there are five distinct states of cluster synchronization, where for each state (panel), the blue squares signify complete synchronization between patches *i* and *j* with *c*_*ij*_ = 1, and the yellow squares are amplitude desynchronized pairs with *c*_*ij*_ = 0. For example, for the leftmost panel, the amplitude-synchronized pairs are (5,7), (4,8), (3,9) and (2,10), which correspond to the time series in Fig. 1(b) and (c). Examining the network structure in Fig. 1(a), we see that this state of cluster synchronization is induced by a specific reflection symmetry: one whose axis of symmetry is the line connecting nodes 1 and 6. In fact, each of the four other distinct cluster-synchronization states is generated by a different reflection symmetry of the network, with their symmetry axes being (4,9), (5,10), (3,8) and (2,7), respectively. The bottom panel in Fig. 2 shows the evolution of *c*_*ij*_ in a long time interval of approximately 15 000 average periods, where the ordinate specifies the position of the matrix element *c*_*ij*_. Note that, because of the symmetry of the matrix and the trivial diagonal elements, only the elements in the upper triangular part of the matrix are shown. To be specific, the position index of *c*_*ij*_ (with *j* > *i*) is calculated as $I=(j-i)+\sum _{i^{\prime }=1}^{i-1}\sum _{j^{\prime }=i^{\prime }+1}^N 1$. There are in total 45 positions in the bottom panel of Fig. 2. In the course of time evolution, there is intermittent switching of the cluster synchronization state. That is, a cluster synchronization state can sustain but only for a finite amount of time and then becomes unstable, after which a short time interval of desynchronization arises. At the end of the desynchronization epoch, the system evolves spontaneously into a randomly chosen cluster synchronization state that could be distinct from the one before the desynchronization epoch. Figure 2 thus indicates that each possible cluster synchronization state enabled by the network symmetry is transient, and the evolution of cluster synchronization within phase synchronization is intermittent.

**Figure 3.**
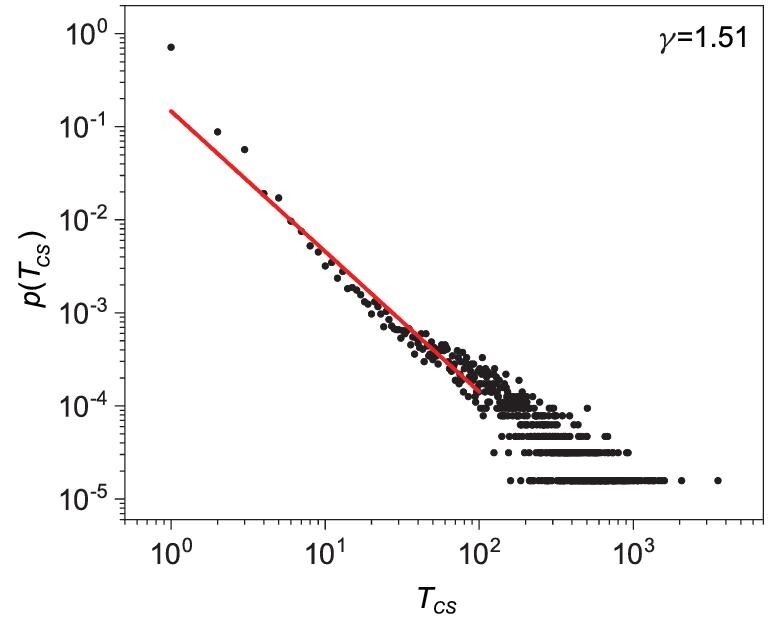
Probability distribution of the transient lifetime—the time for the network to maintain a specific cluster synchronization state. Shown is the probability distribution function *p*(*T*_*CS*_) for ϵ = 0.038, where *T*_*CS*_ denotes the transient lifetime. The distribution can be fitted by an algebraic scaling: $p(T_{C\!\!S})\sim T_{C\!\!S}^{-\gamma }$ with γ ≈ 1.51.

Figure 2 indicates that the time to maintain a specific cluster state, or the transient lifetime denoted as *T*_*CS*_, is irregular. Through Monte Carlo simulation of the network dynamics with a large number of initial conditions, we obtain the probability distribution of *T*_*CS*_, as shown in Fig. 3 for ϵ = 0.038. The distribution is approximately algebraic: $p(T_{C\!S})\sim T_{C\!S}^{-\gamma }$ with the exponent γ ≈ 1.51. The algebraic distribution indicates that an arbitrarily long transient of cluster synchronization can occur with a nonzero probability and, because the value of the exponent is between one and two, the average transient lifetime diverges.

### Dynamical mechanism of intermittency—effect of noise

The five distinct cluster synchronization states enabled by the symmetries of the network, as demonstrated in Fig. 2, are coexisting asymptotic states (or attractors) of the system. That is, the ecological network (1) exhibits multistability, a ubiquitous phenomenon in nonlinear dynamical systems [35–43]. The numerically observed behavior of intermittency in Fig. 2 is effectively random hopping among the coexisting attractors induced by computational ‘noise.’ To see this, consider the regime of the coupling parameter where the cluster synchronization state is weakly stable (to be defined precisely below) and imagine simulating the system dynamics using an infinitely accurate algorithm on an ideal machine with zero round-off error. In this idealized setting, from a given set of initial conditions, the system dynamics will approach an attractor corresponding to a specific cluster synchronization state. Because of absence of error or noise of any sort, the system will remain in this attractor indefinitely. Realistically, inevitable random computational errors will ‘kick’ the system out of the attractor and settle it into another attractor corresponding to a different cluster synchronization state but for a finite amount of time, kick it out again, and so on, generating an intermittent hopping or switching behavior as demonstrated in Fig. 2.

**Figure 4.**
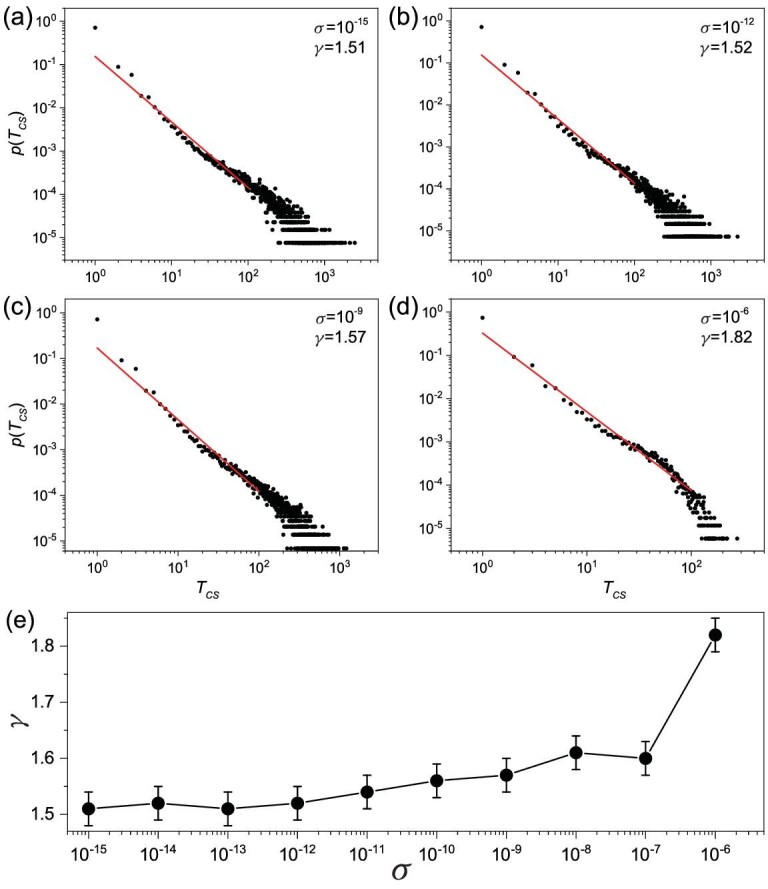
Effect of noise on the algebraic distribution of the transient lifetime of the cluster synchronization state. (a)–(d) Algebraic distribution *p*(*T*_*CS*_) for four values of the noise amplitude σ: 10^−15^, 10^−12^, 10^−9^ and 10^−6^. The values of the algebraic exponent are approximately 1.51, 1.52, 1.57 and 1.82, respectively. Larger noise reduces (often significantly) the probability of a long transient lifetime. (e) An increasing trend of the algebraic exponent γ with noise amplitude σ.

To provide support for this mechanism of intermittency, we investigate the effect of deliberately supplied noise on intermittency. In particular, we assume that system equation (1) is subject to additive, independent, Gaussian white noise η(*t*) at each node for each dynamical variable (*x*, *y*, or $z$), with 〈η(*t*)〉 = 0 and 〈η(*t*)η(*t*^′^)〉 = σ^2^δ(*t* − *t*^′^), where σ is the noise amplitude and δ(*x*) is the Dirac delta function. We calculate the distributions of the transient lifetime for different noise levels. The idea is that, when the noise amplitude is smaller than or comparable to the computational error (about 10^−15^), the algebraic distribution should be similar to that without external noise with a similar exponent to that in Fig. 3, i.e. about 1.5. Stronger noise will induce more frequent switching and reduce the probability of a long transient time, giving rise to a larger exponent. Evidence for this scenario is presented in Fig. 4, where we observe that a larger noise amplitude indeed leads to a larger value of the algebraic scaling exponent γ. For variation of noise amplitude over nine orders of magnitude (from 10^−15^ to 10^−6^), the lifetime distribution *p*(*T*_*CS*_) remains robustly algebraic, and the value of the algebraic exponent γ increases from about 1.5 to 1.8. For example, for σ = 10^−15^, there are long lifetime intervals over 1000 (average cycles of population oscillation). However, for σ = 10^−6^, no such intervals have been observed.

### Evidence of generality: transient cluster synchronization in the Hastings–Powell model

To demonstrate the generality of the phenomena of transient cluster synchronization and intermittency, we consider the Hastings–Powell model of a chaotic food web network [44]:

(2a) \begin{eqnarray*} \dot{x}_{i} & =& x_{i}(1-x_{i})-f_{1}(x_{i})y_{i}, \end{eqnarray*}

(2b) \begin{eqnarray*} \dot{y}_{i} & =& f_{1}(x_{i})y_{i}-f_{2}(y_{i})z_{i}-d_{1}y_{i}\nonumber\\ &&+\,\varepsilon _y\sum ^{N}_{j=1}a_{ij}(y_{j}-y_{i}), \end{eqnarray*}

(2c) \begin{eqnarray*} \dot{z}_{i} & =& f_{2}(y_{i})z_{i}-d_{2}z_{i}+\varepsilon _z\sum ^{N}_{j=1}a_{ij}(z_{j}-z_{i}).\nonumber\\ \end{eqnarray*}

Here the index *i* = 1, 2, …, *N* denotes the individual patches, *x* is the population of species at the lowest level of the food chain, and *y* and $z$ are the populations of the species that prey on *x* and *y*, respectively. The nonlinear functions *f*_*l*_($w$) are given by *f*_*l*_($w$) = *a*_*l*_$w$/(1 + *b*_*l*_$w$), and the representative parameter values [44] are *a*_1_ = 5.0, *a*_2_ = 0.1, *b*_1_ = 3.0, *b*_2_ = 2.0, *d*_1_ = 0.4 and *d*_2_ = 0.01. (The phenomenon of transient cluster synchronization to be reported also occurs for other parameter values, e.g. when *d*_1_ varies in the interval [0.35, 0.4).) Pairwise linear coupling occurs between the *y* and $z$ variables with the corresponding coupling parameters ϵ_*y*_ and ϵ_$z$_.

**Figure 5.**
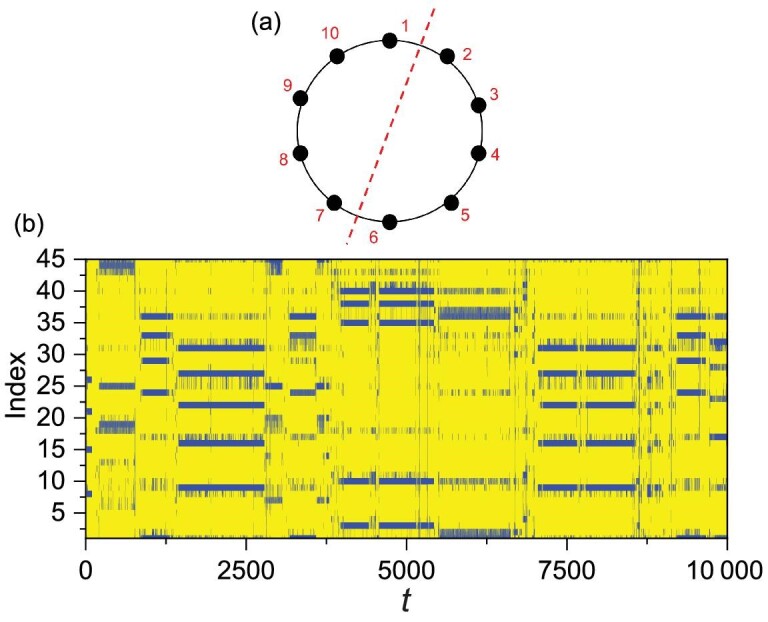
Network structure and intermittent cluster synchronization in the Hastings–Powell model. (a) A dispersal network of ten patches with a regular ring structure. The red dashed line specifies one of the five symmetry axes that lead to five possible cluster synchronization states of patterns. (b) Representative time evolution of the matrix elements *c*_*ij*_(*t*) for ϵ = ϵ_*y*_ = ϵ_$z$_ = 0.00869. The time is rescaled by the average period of the population oscillations.

**Figure 6.**
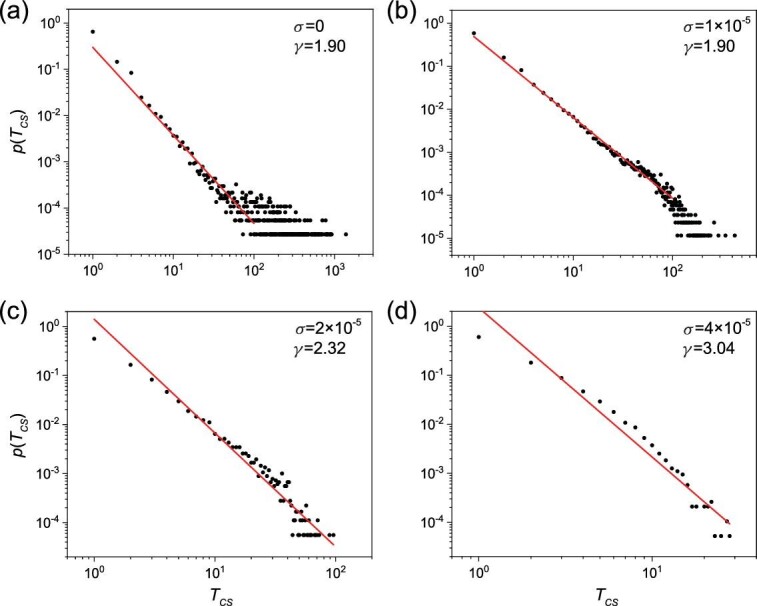
Effect of noise on the algebraic distribution of the transient lifetime of the cluster synchronization state in the Hastings–Powell model. (a)–(d) Algebraic distribution *p*(*T*_*CS*_) for four values of the noise amplitude σ: 0, 1 × 10^−5^, 2 × 10^−5^ and 4 × 10^−5^, respectively.

We study a locally coupled, regular ring network of *n* = 10 patches, as shown in Fig. 5(a). Representative time evolution of the matrix elements *c*_*ij*_ is shown in Fig. 5(b) for ϵ = ϵ_*y*_ = ϵ_$z$_ = 0.00869, where time *t* is rescaled by the average period of the population oscillations. To facilitate observation of cluster synchronization, we define the synchronization matrix element *c*_*ij*_(*t*) as *c*_*ij*_(*t*) = *c*_*ji*_(*t*) = 1 if the difference between the populations $z$ of patches *i* and *j* remains sufficiently small within one natural period *T* of the population oscillation: |$z$_*i*_(*t*) − $z$_*j*_(*t*)| < 2.0 × 10^−2^ for *t* ∈ *T*, and *c*_*ij*_(*t*) = 0 otherwise. Similar to Fig. 2, there is intermittent cluster synchronization in the Hastings–Powell model as well. In Fig. 6 we show the probability distributions of *T*_*CS*_ for different values of the noise amplitude, which are similar to the results in Fig. 4.

## DISCUSSION

Focusing on small, chaotic dispersal networks with relatively strong interactions and a regular structure, we have uncovered a type of transient ecological dynamics in terms of synchronization. In particular, in the parameter regime beyond weak coupling where there is phase synchronization among all the patches but the interactions are not strong enough for global synchronization in both phase and amplitude among all patches, transient amplitude synchronization between the symmetric patches can arise. (Phase synchronization occurs in the regime of weak coupling, yet no cluster phase synchronization has been observed about the transition point.) The emergence of cluster synchronization in amplitude within phase synchronization represents a remarkable organization of synchronous dynamics in ecological networks. Each symmetry in the network structure generates a distinct cluster synchronization pattern. Multiple symmetries in the network lead to multiple coexisting cluster synchronization patterns (attractors). Because of instability and noise, each cluster synchronization pattern can last for a finite amount of time, leading to random, intermittent switching among the coexisting patterns. The transient time during which a particular cluster synchronization pattern can be maintained follows an algebraic probability distribution. General symmetry considerations enable us to define the cluster synchronization manifold and to quantify its stability by calculating the largest transverse Lyapunov exponent (see the Methods section and Section I of the SM). Finite-time fluctuations of this exponent into both the positive and negative sides are key to understanding the intermittent behavior. A strong similarity to random walk dynamics provides a natural explanation of not only the algebraic nature of the transient lifetime distribution but also the value of the algebraic exponent. Alterations in the structure of the network do not affect these results. For example, we have studied a one-dimensional ring network with an odd number of patches and a spatially two-dimensional lattice, and found that the phenomena of cluster synchronization in amplitude shadowed by chaotic phase synchronization and intermittency persist (see Sections IV and V of the SM). In addition, factors such as variations in coupling strength (see Sections II and VI of the SM) and local parameters (see Section VIII of the SM), noise perturbations (see Section VII of the SM), and symmetry perturbations (see Section XIII of the SM) do not significantly alter the phenomenon.

Our stability analysis has revealed the fundamental role played by network symmetry in the emergence of transient cluster synchronization and intermittency. Symmetry considerations can also be used to explain intriguing, counterintuitive synchronization phenomena in ecological networks. For example, in a previous work on a class of dispersal ecological networks, essentially a nondimensional and spatially structured form of the Rosenzweig–MacArthur predator–prey model [45], it was found that the dispersal network structure has a strong effect on the ecological dynamics in that randomizing the structure of an otherwise regular network tends to induce desynchronization with prolonged transient dynamics [34]. This contrasts the result in the literature of complex networks where synchronization is typically favored by creating random shortcuts in a large regular network, i.e. by making the network structure the small-world type [46–48]. The paradox is naturally resolved by resorting to symmetry. In particular, in the small regular network studied in [34], the observed cluster synchronization patterns are the result of the reflection symmetries of the network. Adding random shortcuts destroys certain symmetry and, consequently, the corresponding synchronization pattern.

In realistic ecological networks, both the dynamics of the patches and the interactions among them can be nonidentical. As the formation of synchronous clusters relies on the network symmetry, a natural question is whether transient cluster synchronization can be observed in ecological networks of nonidentical oscillators and heterogeneous interactions. One approach to addressing this is to introduce perturbations, e.g. parameter and coupling perturbations, to the system and to test if transient cluster synchronization persists. Our computations provided an affirmative answer (see Section XIII of the SM). The results are consistent with the previous findings in the physics literature, where stable cluster synchronization persists when the network symmetries are slightly broken or when the oscillator parameters are slightly perturbed [30,49,50]. Besides ecological networks, we have also observed transient cluster synchronization in the network of coupled chaotic Rössler oscillators (see Section IX of the SM), suggesting the generality of the phenomenon. Whether this phenomenon can arise in large-scale complex networks with heterogeneous nodal dynamics is an open question worth pursuing.

The importance of transients in ecological systems has been increasingly recognized [20–25]. Our work has unearthed a type of transient behavior in the collective dynamics of ecological systems: a synchronization pattern can last for a finite amount of time and be replaced by a completely different pattern in a relatively short time. The finding of transient synchronization dynamics may have implications to ecological management and conservation, and provide insights into experimental observations. For instance, in a recent experiment on the planktonic predator–prey system [51], it was shown that, whereas the abundances of the predator and prey display mostly regular and coherent oscillations, short episodes of irregular and noncoherent oscillations can arise occasionally, making the system switch randomly among different patterns. Furthermore, controlled experiments and simulation of the mathematical model suggest that the switching behavior can be attributed to the intrinsic stochasticity of the system dynamics. The switching behavior reported in [51] is quite similar to the phenomenon of transient, intermittent cluster synchronization uncovered here. As pointed out in [52], the key to explaining the experimentally observed phenomenon is to uncover the role of transient dynamics—the main question that has been addressed in our present work. The findings reported provide fresh insights into the recent experimental results in [51], and we anticipate that the findings will help interpret future experimental results not only in ecological systems, but also in biological, neuronal and physical systems where the system dynamics are represented by complex networks of coupled nonlinear oscillators and pattern switching plays a key role in the system functions.

## METHODS

**Figure 7.**
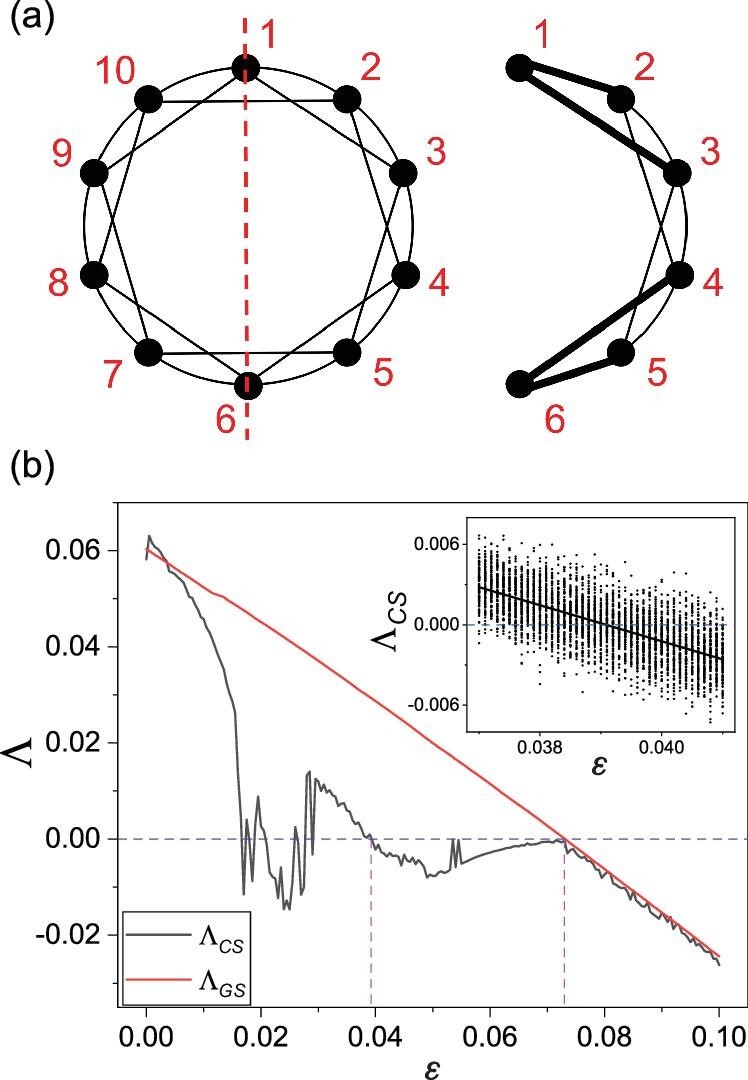
Network symmetry and conditional Lyapunov exponent determining the stability of cluster synchronization. (a) The original (left) and reduced network (right). The red dashed line specifies one of the symmetry axes. The reduced network is weighted, where the thickness of an edge indicates the corresponding weight. (b) The conditional Lyapunov exponent Λ_*CS*_ quantifying the stability of cluster synchronization versus ϵ (the gray curve). The transverse Lyapunov exponent Λ_*GS*_ characterizes the stability of global synchronization (the red curve). Both exponents are calculated using a long time interval (10^5^). The pink vertical dashed line at $\varepsilon \approx 0.039\equiv \varepsilon ^{C\!\!S}_c$ is the critical coupling above which the cluster synchronization is stable, while that at $\varepsilon \approx 0.073 \equiv \varepsilon ^{G\!\!S}_c$ is the transition point to stable global synchronization. The inset shows the values of Λ_*CS*_ calculated in finite time (10^3^) with 100 realizations, and the solid black line is the linear fit of the data points. When the coupling parameter is in the vicinity of $\varepsilon ^{C\!\!S}_c$, intermittent cluster synchronization can emerge. For $\varepsilon \lesssim \varepsilon ^{C\!\!S}_c$, because Λ_*CS*_ is slightly positive, intermittency can be observed without external noise (cf. Fig. 2). For $\varepsilon \gtrsim \varepsilon ^{C\!\!S}_c$, because of the negativity of Λ_*CS*_, cluster synchronization is stable but intermittency can still arise when there is external noise of reasonably large amplitude.

The stability of the cluster synchronization states can be analyzed by means of the conditional Lyapunov exponent. The key to the emergence of cluster synchronization lies in the symmetry of the network, based on which the original network can be reduced [53]. In Fig. 7(a) we present one example, where the symmetry axis is the line connecting nodes 1 and 6 in the original network (the left panel). In this case, the four nodes on the left-hand side of the symmetry axis are equivalent to their respective mirror counterparts on the right-hand side, generating four pairs (clusters) of synchronous nodes: 2 and 10, 3 and 9, 4 and 8, as well as 5 and 7. The network is thus equivalent to a reduced network with six independent nodes, as shown in the right panel of Fig. 7(a), where the edges in the reduced network are weighted [53]. The reduced network defines the dynamics of the synchronization manifold

(3) \begin{equation*} \dot{\mathbf {X}} = \mathbf {F} + \varepsilon \mathcal {M}\cdot \mathbf {H}, \end{equation*}

where $\mathcal {M}$ is the coupling matrix of the reduced network, $\mathbf {X}$, $\mathbf {F}$ and $\mathbf {H}$ are respectively the state vector, the velocity fields of isolated nodal dynamics and the coupling function.

Let $\delta \mathbf {X}$ be infinitesimal perturbations transverse to the cluster synchronization manifold, whose evolution is governed by the variational equation

(4) \begin{equation*} \delta \dot{\mathbf {X}} = (\mathcal {DF} + \varepsilon \mathcal {L}\cdot \mathcal {DH})\cdot \delta \mathbf {X}, \end{equation*}

where $\mathcal {L}$ is the transverse Laplacian matrix, and $\mathcal {DF}$ and $\mathcal {DH}$ are the Jacobian matrices of the isolated nodal dynamics and of the coupling function, respectively. Combining equations (3) and (4), we can calculate the largest transverse Lyapunov exponent Λ_*CS*_ (or the conditional Lyapunov exponent), which depends on the coupling parameter ϵ. The necessary condition for the cluster synchronous state to be stable is Λ_*CS*_ < 0. In Fig. 7(b) we show Λ_*CS*_ as a function of ϵ (the solid gray curve). Also shown is the transverse Lyapunov exponent Λ_*GS*_ determining the stability of global synchronization (solid red curve). The wild fluctuations of Λ_*CS*_ in the interval ϵ ∈ (0.015, 0.03) are due to the occurrence of periodic windows together with transient chaos [54]. Transition to stable cluster synchronization occurs at $\varepsilon \approx 0.039\equiv \varepsilon _c^{C\!S}$, and transition to global (phase and amplitude) synchronization occurs at $\varepsilon \approx 0.073 \equiv \varepsilon _c^{G\!S}$.

For $\varepsilon \lesssim \varepsilon _c^{C\!S}$, cluster synchronization is asymptotically unstable. However, there are epochs of time during which the synchronous dynamics are stable, as indicated by the spread in the values of the conditional Lyapunov exponent calculated in finite time (e.g. 10^3^) into the negative side, as can be seen from the inset in Fig. 7(b). For ϵ = 0.038, the asymptotic value of Λ_*CS*_ is close to zero. The probabilities for the value of the finite-time exponent Λ_*CS*_(*t*) to be positive and negative are thus approximately equal. The dynamics of cluster synchronization can then be treated as an unbiased random walk. For such a stochastic process, the distribution of the first passage time [55] is algebraic with scaling exponent 1.5, which explains the scaling exemplified in Fig. 3. When external noise is present, the underlying random walk process becomes biased. In this case, the scaling exponent of the transient cluster synchronization time deviates from 1.5, as demonstrated in Fig. 4.

A full description of the methods is given in Section II of the SM.

## DATA AVAILABILITY

All relevant data are available from the authors upon request.

## CODE AVAILABILITY

All relevant computer codes are available from the authors upon request.

## Supplementary Material

nwaa269_Supplemental_File
