# Supplementary material for: Synchronization within synchronization: transients and intermittency in ecological networks
Source: Natl Sci Rev. 2020 Oct 24;8(10):nwaa269. doi: 10.1093/nsr/nwaa269 (PMC8566182; doi:10.1093/nsr/nwaa269)
Supplement: nwaa269_Supplemental_File [file nwaa269_supplemental_file.pdf]

Supplementary Information for

Synchronization within synchronization: transients and intermittency in ecological networks

Huawei Fan, Ling-Wei Kong, Xingang Wang, Alan Hastings, and Ying-Cheng Lai*

CONTENTS

I. Stability analysis of cluster synchronization 2

II. Cluster and global synchronization for nonidentical coupling 4

III. Fluctuations of the finite time Lyapunov exponent 4

IV. Transients and intermittent synchronization in a network of odd number of patches 6

V. Transients and intermittent synchronization in a two-dimensional lattice of patches 7

VI. Effect of coupling on transients and intermittency 8

VII. Effect of noise on transients and intermittency for stronger coupling 9

VIII. Transient cluster synchronization for alternative values of the local parameters 10

IX. Transient cluster synchronization in coupled chaotic Rössler oscillators 11

X. Inverse cumulative distribution of transient lifetime 12

XI. Variation of degree of synchronization about ε_c^{CS} 13

XII. Effect of coupling on statistical properties of synchronization manifold 14

XIII. Effect of symmetry perturbations on transient behaviors 16

References 16

I. STABILITY ANALYSIS OF CLUSTER SYNCHRONIZATION

Let $\mathcal{S}$ be the permutation symmetry that the network possesses and $\mathbf{X}_s$ be a vector in the cluster synchronization manifold associated with $\mathcal{S}$. The dynamics of $\mathbf{X}_s$ are governed by

$$\frac{d\mathbf{X}_s}{dt} = \mathbf{F}(\mathbf{X}_s) + \varepsilon \mathcal{M} \cdot \mathbf{H}(\mathbf{X}_s), \tag{S1.1}$$

where $\mathbf{F}(\mathbf{x})$ is the velocity field of the isolated nodal dynamics, $\mathbf{H}(\mathbf{x})$ is the coupling function, and $\mathcal{M}$ is the coupling matrix of the reduced network. The stability of the cluster synchronization manifold is determined by the variational equation

$$\frac{d\delta\mathbf{X}}{dt} = [\mathcal{DF}(\mathbf{X}_s) + \varepsilon \mathcal{L} \cdot \mathcal{DH}(\mathbf{X}_s)] \cdot \delta\mathbf{X}, \tag{S1.2}$$

where $\delta\mathbf{X}$ is an infinitesimal perturbation transverse to the manifold, $\mathcal{L}$ is the transverse matrix determined by the network symmetry, $\mathcal{DF}(\mathbf{X}_s)$ and $\mathcal{DH}(\mathbf{X}_s)$ are the Jacobian matrices of the velocity field and of the coupling function evaluated at $\mathbf{X}_s$, respectively. For the cluster synchronization state to be stable, the necessary condition is that $\delta\mathbf{X}$ approaches zero exponentially with time. Let Λ be the largest Lyapunov exponent calculated from Eq. (S1.2). The stability condition is $\Lambda < 0$.

The coupling matrix $\mathcal{M}$ is constructed according to the network symmetry $\mathcal{S}$, as follows. Assume the network contains n symmetric nodal pairs (the symmetric group) and m "isolated" nodes that are not connected with any node in the symmetric group. The number of nodes in the reduced network is $N' = n + m$, in which the coupling strength that node l receives from node k can be written as

$$\mathcal{M}_{lk} = (\sum_{i \in v_l} \sum_{j \in v_k} a_{ij})/q,$$

where v_l (or v_k) is the set of symmetric nodes in the original network which are represented by node l (or k) in the reduced network, $\mathcal{A} = \{a_{ij}\}$ is the adjacency matrix of the original network, and $q = 2$ (or $q = 1$) if node i belongs to a symmetric pair (or is an isolated node). The transverse matrix $\mathcal{L}$ in Eq. (S1.2) is obtained by transforming the matrix $\mathcal{A}$ into the space spanned by the eigenvectors of the network symmetry matrix $\mathcal{P} = \{p_{ij}\}$, as follows. If nodes i and j are symmetric in the network, we set $p_{ij} = p_{ji} = 1$; if node i is isolated, we set $p_{ii} = 1$; the remaining elements are all set as zero. Let $\mathcal{T}$ be the transformation matrix constructed from the eigenvectors of $\mathcal{P}$, which can be applied to the coupling matrix $\mathcal{G} = \mathcal{A} - \mathcal{K}$ to yield a matrix in the blocked form:

$$\mathcal{G}' = \mathcal{T}^{-1} \cdot \mathcal{G} \cdot \mathcal{T} = \begin{pmatrix} \mathcal{B} & 0 \\ 0 & \mathcal{L} \end{pmatrix}, \tag{S1.3}$$

where $\mathcal{K}$ is the diagonal matrix with elements being the degree $k_{ii} = \sum_j a_{ij}$ of node i, $\mathcal{B}$ characterizes the dynamics in the synchronization manifold (which is transformed from the coupling matrix of the reduced network, $\mathcal{M}$), and $\mathcal{L}$ is the transverse matrix that we set out to find. A straightforward way to distinguish $\mathcal{L}$ from $\mathcal{B}$ is to check which matrix gives the null eigenvalue: $\mathcal{B}$ has a null eigenvalue while $\mathcal{L}$ does not.

For the network shown in Fig. 1(a) in the main text, the symmetry axis is the line connecting nodes 1 and 6, and the four nodes on the left side of the symmetry axis are equivalent to their respective mirror counterparts on the right side, generating four pairs (clusters) of synchronous

nodes: 2 and 10, 3 and 9, 4 and 8, as well as 5 and 7. Hence, the cluster synchronization manifold is defined by $\mathbf{x}_1 \equiv \mathbf{x}_1^s$, $\mathbf{x}_2 = \mathbf{x}_{10} \equiv \mathbf{x}_{2,10}^s$, $\mathbf{x}_3 = \mathbf{x}_9 \equiv \mathbf{x}_{3,9}^s$, $\mathbf{x}_4 = \mathbf{x}_8 \equiv \mathbf{x}_{4,8}^s$, $\mathbf{x}_5 = \mathbf{x}_7 \equiv \mathbf{x}_{5,7}^s$, and $\mathbf{x}_6 \equiv \mathbf{x}_6^s$ where $\mathbf{x}_i$ is the vector of the dynamical variables of node i. More specifically, the vector $\mathbf{X}_s$ in the synchronization manifold, the velocity field $\mathbf{F}$ and the coupling function $\mathbf{H}$ in Eq. (S1.1) are

$$\begin{aligned}
\mathbf{X}_s &= [(\mathbf{x}_1^s)^T, (\mathbf{x}_{2,10}^s)^T, (\mathbf{x}_{3,9}^s)^T, (\mathbf{x}_{4,8}^s)^T, (\mathbf{x}_{5,7}^s)^T, (\mathbf{x}_6^s)^T]^T, \\
\mathbf{F} &= [(\mathbf{F}(\mathbf{x}_1^s))^T, (\mathbf{F}(\mathbf{x}_{2,10}^s))^T, (\mathbf{F}(\mathbf{x}_{3,9}^s))^T, (\mathbf{F}(\mathbf{x}_{4,8}^s))^T, (\mathbf{F}(\mathbf{x}_{5,7}^s))^T, (\mathbf{F}(\mathbf{x}_6^s))^T]^T, \\
\mathbf{H} &= [(\mathbf{H}(\mathbf{x}_1^s))^T, (\mathbf{H}(\mathbf{x}_{2,10}^s))^T, (\mathbf{H}(\mathbf{x}_{3,9}^s))^T, (\mathbf{H}(\mathbf{x}_{4,8}^s))^T, (\mathbf{H}(\mathbf{x}_{5,7}^s))^T, (\mathbf{H}(\mathbf{x}_6^s))^T]^T,
\end{aligned}$$

In the cluster synchronization state, the dynamics of the network are equivalent to the dynamics of a reduced network with $N' = 6$ independent nodes, as shown in the right panel of Fig. 7(a) in the main text. The coupling matrix of the reduced network is

$$\mathcal{M} = \begin{bmatrix} -4 & 2 & 2 & 0 & 0 & 0 \\ 1 & -3 & 1 & 1 & 0 & 0 \\ 1 & 1 & -4 & 1 & 1 & 0 \\ 0 & 1 & 1 & -4 & 1 & 1 \\ 0 & 0 & 1 & 1 & -3 & 1 \\ 0 & 0 & 0 & 2 & 2 & -4 \end{bmatrix}. \tag{S1.4}$$

The permutation matrix associated with the network symmetry is

$$\mathcal{P} = \begin{bmatrix} 1&0&0&0&0&0&0&0&0&0 \\ 0&0&0&0&0&0&0&0&0&1 \\ 0&0&0&0&0&0&0&0&1&0 \\ 0&0&0&0&0&0&0&1&0&0 \\ 0&0&0&0&0&0&1&0&0&0 \\ 0&0&0&0&0&1&0&0&0&0 \\ 0&0&0&0&1&0&0&0&0&0 \\ 0&0&0&1&0&0&0&0&0&0 \\ 0&0&1&0&0&0&0&0&0&0 \\ 0&1&0&0&0&0&0&0&0&0 \end{bmatrix}. \tag{S1.5}$$

Transforming the coupling matrix of the original network to the space spanned by the eigenvectors of $\mathcal{P}$, we obtain the transverse matrix

$$\mathcal{L} = \begin{bmatrix} -5 & 1 & 1 & 0 \\ 1 & -4 & 1 & 1 \\ 1 & 1 & -4 & 1 \\ 0 & 1 & 1 & -5 \end{bmatrix}, \tag{S1.6}$$

which is used in Eq. (S1.2) for calculating the conditional Lyapunov exponent Λ plotted in Fig. 7 in the main text.

Altogether, in addition to the case shown in Fig. 1(a) in the main text, the network has four other reflection symmetries, with axes being the lines connecting nodal pairs $(4,9)$, $(5,10)$, $(3,8)$, and $(2,7)$, respectively, each generating a distinct pattern of cluster synchronization, as demonstrated in Fig. 2 in the main text. The stability of the corresponding synchronization manifold can be analyzed in a similar manner.

II. CLUSTER AND GLOBAL SYNCHRONIZATION FOR NONIDENTICAL COUPLING

We study the general case where the coupling parameters associated with herbivores and predators are not identical: $\varepsilon_y \neq \varepsilon_z$. We focus on the values of the two transverse Lyapunov exponents: Λ_{CS} and Λ_{GS}, which determine the stability of cluster and global synchronization in the network, respectively. Figure S1(a) shows the color coded values of Λ_{CS} in the parameter plane $(\varepsilon_y, \varepsilon_z)$, where the value of Λ_{CS} mostly decreases with ε_y and varying the value of ε_z has little effect on the exponent. For instance, the black curve representing the contour of $\Lambda_{CS} = 0$ and therefore separating the synchronization and desynchronization regions is almost vertical and located about $\varepsilon_y \approx 0.04$. This behavior implies that the coupling among the herbivores is more important than that among the predators for cluster synchronization. For global synchronization, it is convenient to use the generalized coupling parameters: $K_y = \lambda\varepsilon_y$ and $K_z = \lambda\varepsilon_z$, where λ is the eigenvalue of the Laplacian matrix of the network. The color coded values of Λ_{GS} are shown in Fig. S1(b). It can be seen that the coupling among the herbivores also plays an important role in global synchronization of the whole network.

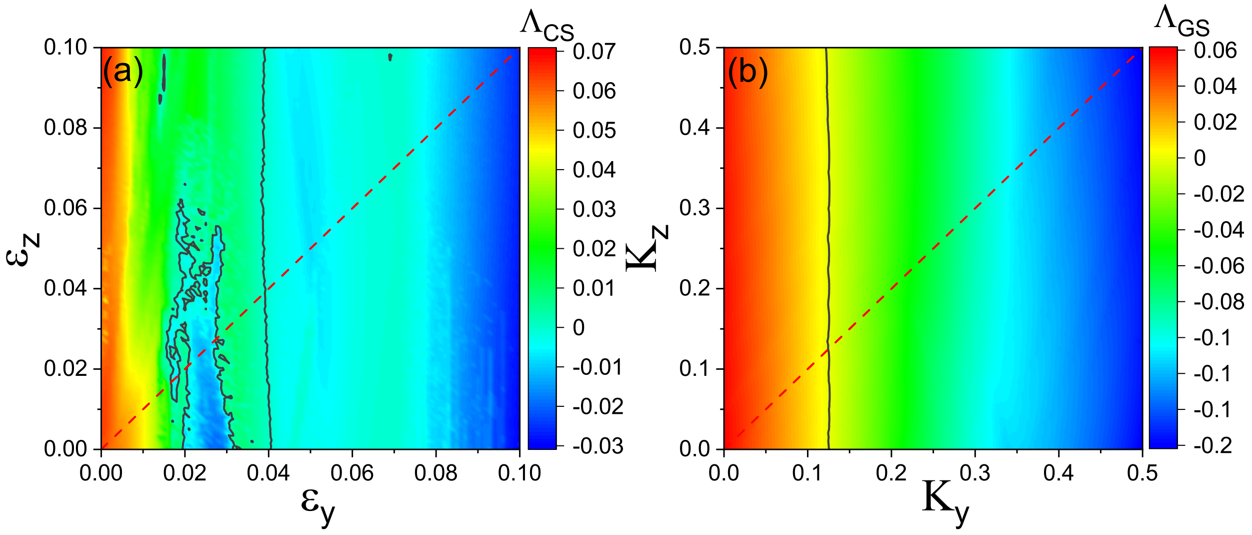

FIG. S1. *Cluster and global synchronization for nonidentical values of the coupling parameters associated with herbivores and predators.* (a) Values of the conditional Lyapunov exponent Λ_{CS} characterizing cluster synchronization in the parameter plane $(\varepsilon_y, \varepsilon_z)$, and (b) values of the transverse Lyapunov exponent Λ_{GS} for global synchronization (in both phase and amplitude) in the plane of the generalized coupling parameters (K_y, K_z). In each panel, the mostly vertical black curve is the contour along which the value of the corresponding exponent is zero.

III. FLUCTUATIONS OF THE FINITE TIME LYAPUNOV EXPONENT

The top panel of Fig. S2 demonstrates explicitly the fluctuations of the finite time Lyapunov exponent for $\varepsilon = 0.038$, where the exponent Λ_{CS} is calculated in a short time interval $\Delta t = 10^{-2}$ so that it can be regarded as a continuous function of time. For reference, the corresponding time evolution of all species populations in the network are shown (in the three lower panels). It can

be seen that, in any one cycle of population oscillation, Λ_{CS} possesses both positive and negative values, making its asymptotic value approximately zero. In this case, cluster synchronization can be maintained but for a finite amount of time - arbitrarily small uncertainties or perturbations (e.g., inevitable computational errors) can drive the network out of the specific cluster synchronization state and make it approach another coexisting state, generating intermittency as demonstrated in Fig. 2 in the main text.

A noticeable feature of the finite time conditional Lyapunov exponent $\Lambda_{CS}(t)$, as shown in Fig. S2, is that its negative peaks are relatively sharp and each corresponds to the position of a near maximum for $y_i(t)$ and $z_i(t)$, as indicated by the vertical red dashed lines in Fig. S2. This indicates that the system tends to synchronize when the herbivore and predator populations begin to decay.

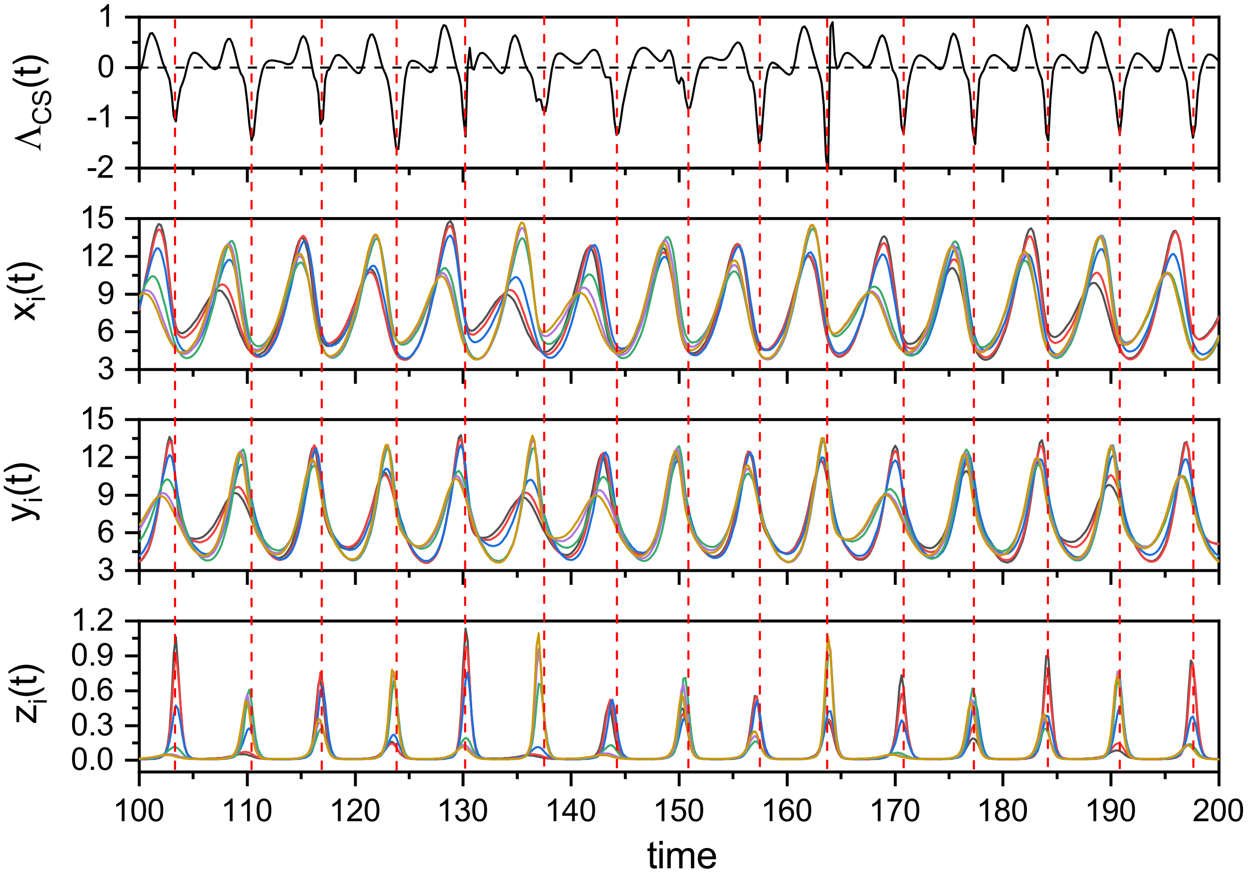

FIG. S2. *Time evolution of the finite time conditional Lyapunov exponent* $\Lambda_{CS}(t)$. The four panels (from top down) correspond to $\Lambda_{CS}(t)$ and the evolution of species populations x_i, y_i, and z_i in all patches (distinguished by different colors). The coupling parameter is $\varepsilon = 0.038$ - the same value as in Fig. 2 in the main text.

IV. TRANSIENTS AND INTERMITTENT SYNCHRONIZATION IN A NETWORK OF ODD NUMBER OF PATCHES

We consider a spatial network of $n = 11$ patches. Different from the case of an even number of patches, here the network has 6 symmetry axes and, for each axis, there are 5 symmetric nodal pairs and one isolated node (node 1). The upper panel of Fig. S3 shows the network structure. Representative time evolution of the matrix elements c_{ij} is shown in the lower panel of Fig. S3 (for $\varepsilon = 0.05$). As in the network of an even number of patches treated in the main text, the phenomena of cluster synchronization shadowed by chaotic phase synchronization and intermittency persist for networks with an odd number of patches.

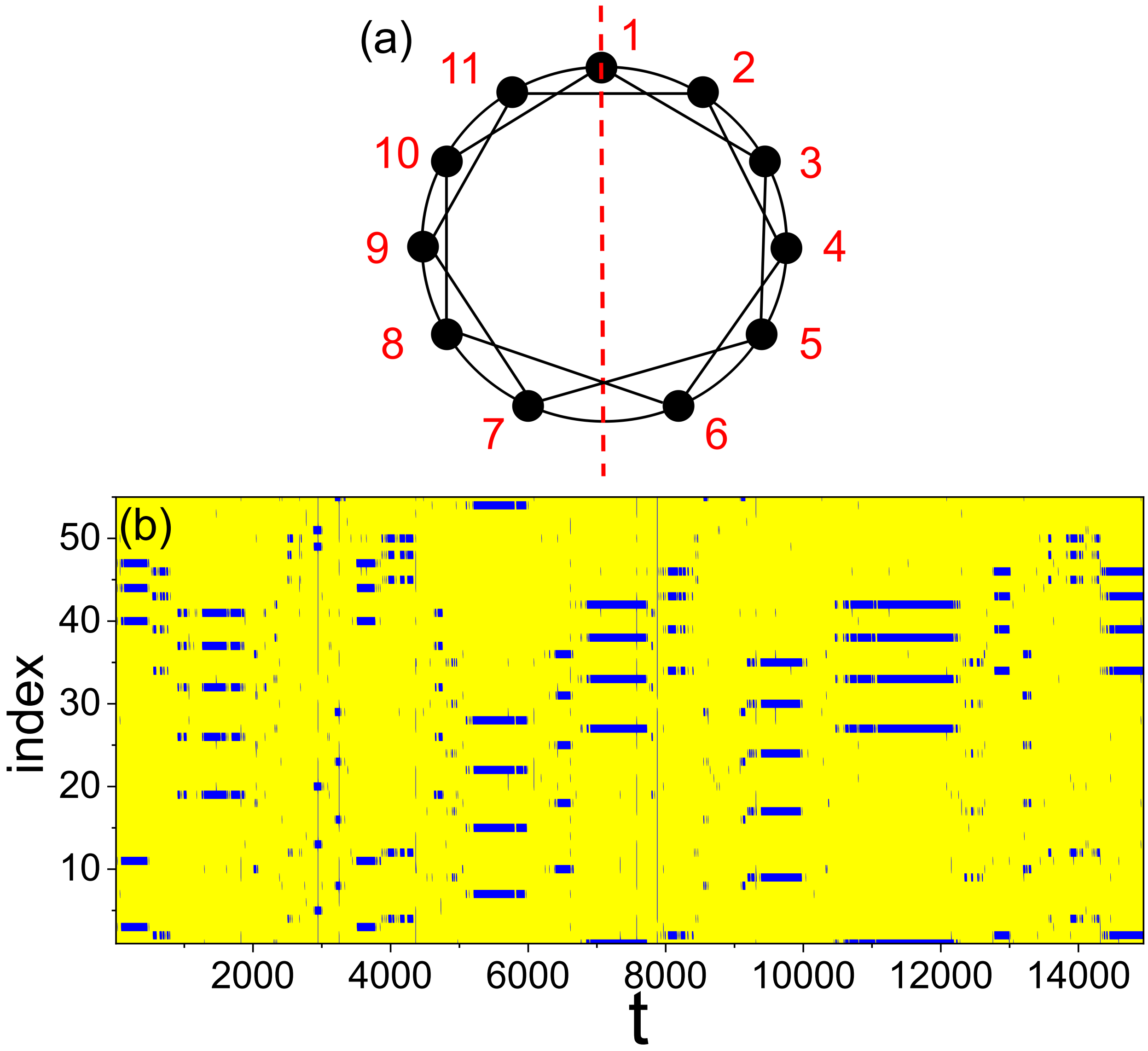

FIG. S3. *Structure and intermittent cluster synchronization in a network of odd number of patches.* (a) A dispersal network of eleven patches with a regular ring structure. The red dotted line specifies one of the symmetry axes. (b) Time evolution of the matrix elements $c_{ij}(t)$ for $\varepsilon = 0.05$.

V. TRANSIENTS AND INTERMITTENT SYNCHRONIZATION IN A TWO-DIMENSIONAL LATTICE OF PATCHES

We study a two-dimensional lattice of patches, as shown in the upper panel of Fig. S4. The network size is $n = 16$ with periodic boundary conditions. The lower panel in Fig. S4 shows the time evolution of the matrix elements c_{ij} in a long time interval of approximately 60000 average periods. In spite of the spatially two-dimensional structure of the network, the phenomena of cluster synchronization umbrellaed by chaotic phase synchronization and intermittent switching among distinct cluster synchronization patterns still occur.

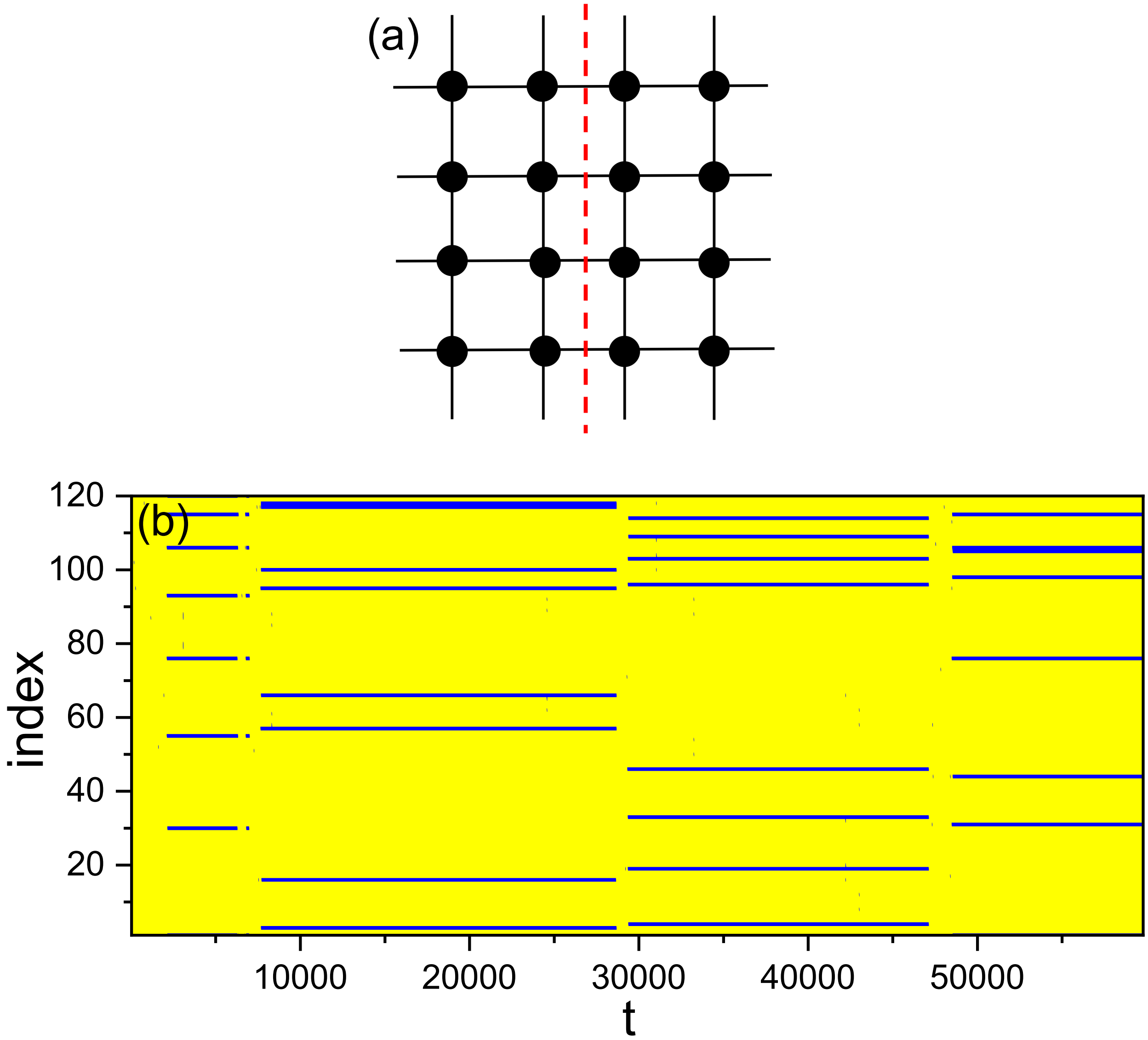

FIG. S4. *Network structure and intermittent cluster synchronization in a two-dimensional spatial lattice of patches*. (a) A dispersal network of sixteen patches with a two-dimensional lattice structure. The red dotted line specifies one of the symmetry axes. (b) The time evolution of the matrix elements $c_{ij}(t)$ for $\varepsilon = 0.0257$.

VI. EFFECT OF COUPLING ON TRANSIENTS AND INTERMITTENCY

How does the value of the coupling parameter ε affect transient cluster synchronization and intermittency? To address this question, we calculate the evolution of the cluster synchronization matrix for a systematic set of ε values. Figure S5 shows four cases: $\varepsilon = 0.03, 0.035, 0.04$ and 0.045. For $\varepsilon \lesssim 0.03$, cluster synchronization is rare, which becomes more frequent as the value of ε is increased from 0.03. For $\varepsilon \gtrsim 0.045$, the duration of cluster synchronization becomes long: a particular state can last for a long time and numerically it becomes difficult to obtain intermittency. Nonetheless, the phenomenon of intermittent cluster synchronization can occur in a finite interval of the coupling parameter. Especially, for system (1) in the main text, the parameter interval is $0.035 \lesssim \varepsilon \lesssim 0.04$.

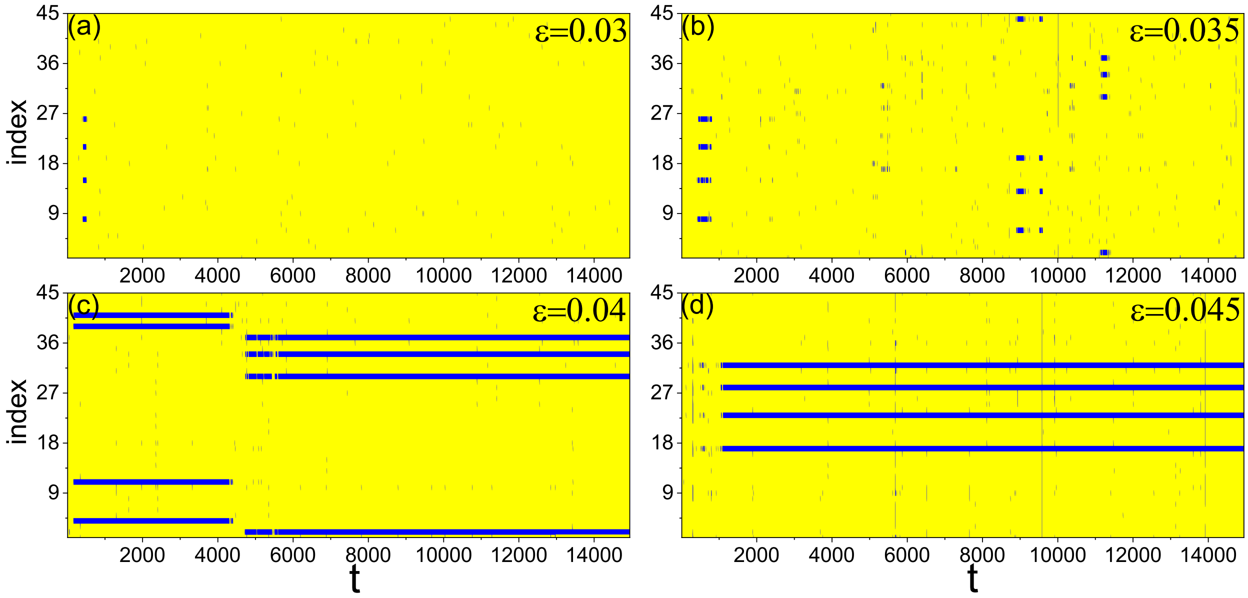

FIG. S5. *Effect of coupling on cluster synchronization.* Shown is the time evolution of the elements of the cluster synchronization matrix $c_{ij}(t)$ for (a) $\varepsilon = 0.03$, (b) $\varepsilon = 0.035$, (c) $\varepsilon = 0.04$, and (d) $\varepsilon = 0.045$. For the networked system (1) in the main text, the phenomenon of intermittent cluster synchronization occurs for $0.035 \lesssim \varepsilon \lesssim 0.04$.

VII. EFFECT OF NOISE ON TRANSIENTS AND INTERMITTENCY FOR STRONGER COUPLING

We calculate the distributions of the transient lifetime T_{CS} in a stronger coupling regime for four values of the noise amplitude, as shown in Fig. S6. In general, strong coupling leads to a longer transient lifetime, giving rise to smaller values of the algebraic exponent. For example, for $\sigma = 10^{-9}$, the exponent is $\gamma \approx 1.57$ for $\varepsilon = 0.038$. For $\varepsilon = 0.04$, the exponent has the value $\gamma \approx 1.41$.

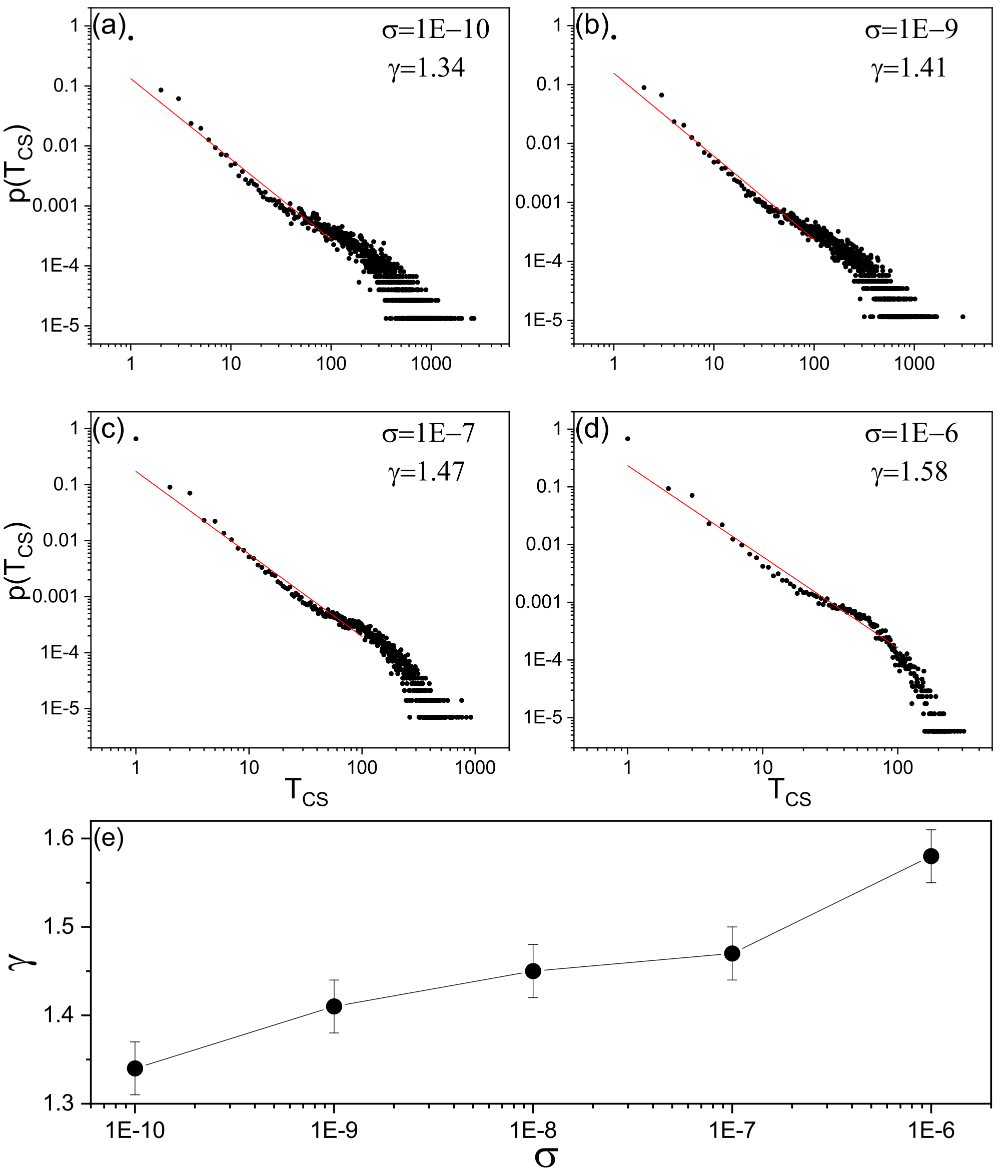

FIG. S6. *Effect of noise on algebraic distribution of the transient lifetime of cluster synchronization state.* (a-d) For $\varepsilon = 0.04$, algebraic distribution $p(T_{CS})$ for four values of noise amplitude σ: 10^{-10}, 10^{-9}, 10^{-7}, and 10^{-6}. The values of the algebraic exponent are approximately 1.34, 1.41, 1.47, and 1.58, respectively. (e) The algebraic exponent γ versus the noise amplitude σ.

VIII. TRANSIENT CLUSTER SYNCHRONIZATION FOR ALTERNATIVE VALUES OF THE LOCAL PARAMETERS

The phenomena reported in the main text have also been observed for alternative values of the parameters of the local dynamics. For example, changing the parameter b in the chaotic food web system [Eq. (1) in the main text] to 0.9 gives Fig. S7(a), the time evolution of the dynamical variables. It can be seen that, similar to the results in the main text [Fig. 2], the time evolution is characteristic of the phenomenon of intermittent cluster synchronization. This is also the case for the Hastings-Powell system. In particular, setting $d_1 = 0.35$ and $\varepsilon = 0.025$ in Eq. (2) in the main text, we obtain Fig. S7(b), the time evolution of the dynamical variables, where intermittent cluster synchronization occurs.

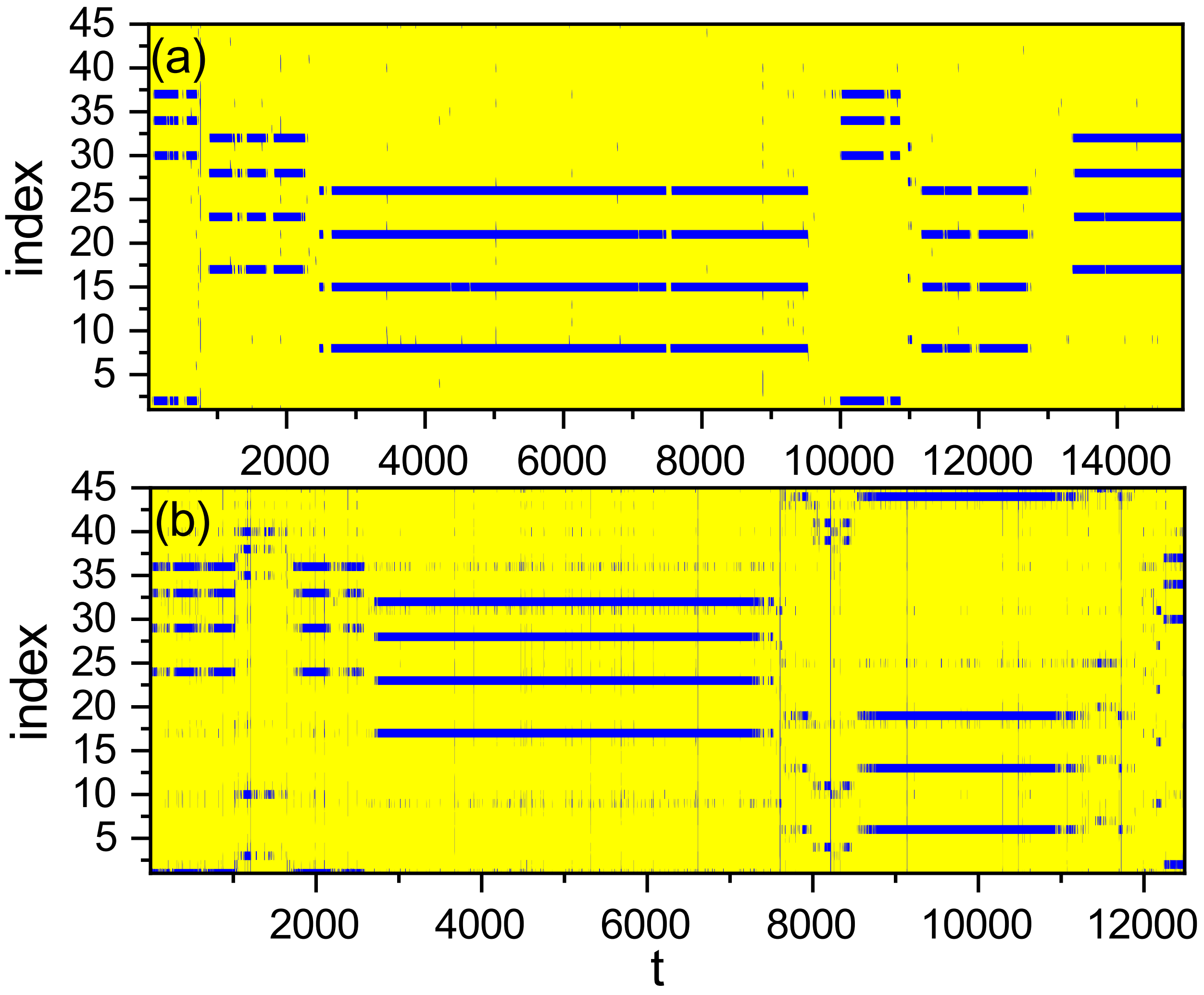

FIG. S7. *Transient cluster synchronization for alternative values of the parameters of the local nodal dynamics.* Shown is the time evolution of the dynamical variables for (a) the chaotic food web model for $b = 0.9$, and (b) the Hastings-Powell system for $d_1 = 0.35$ and $\varepsilon = 0.025$. Other parameter values are the same as those in the main text.

IX. TRANSIENT CLUSTER SYNCHRONIZATION IN COUPLED CHAOTIC RÖSSLER OSCILLATORS

Transient cluster synchronization has also been observed in networks of coupled chaotic Rössler oscillators. The network structure is identical to that in Fig. 1(a) in the main text, with the local dynamical system replaced by the classical chaotic Rössler oscillator. The network dynamical equations are

$$\begin{aligned}\dot{x}_i &= -y_i - z_i,\\ \dot{y}_i &= x_i + ay_i + \varepsilon \sum_{j=1}^{N} a_{ij}(y_j - y_i),\\ \dot{z}_i &= z_i(x_i - c) + b,\end{aligned}$$

where $i, j = 1, \ldots, N$ are the nodal indices, a_{ij} are the elements of the network adjacency matrix [Fig. 1(a) in the main text], and ε is the uniform coupling strength. For $(a,b,c) = (0.2, 0.2, 5.7)$, the oscillator generates a chaotic attractor. Figure S8 shows, for $\varepsilon = 0.046$, the time evolution of the synchronization relationship among the nodal dynamics. Similar to the results from the coupled ecological oscillators, the system switches randomly among different cluster synchronization states during the course of system evolution.

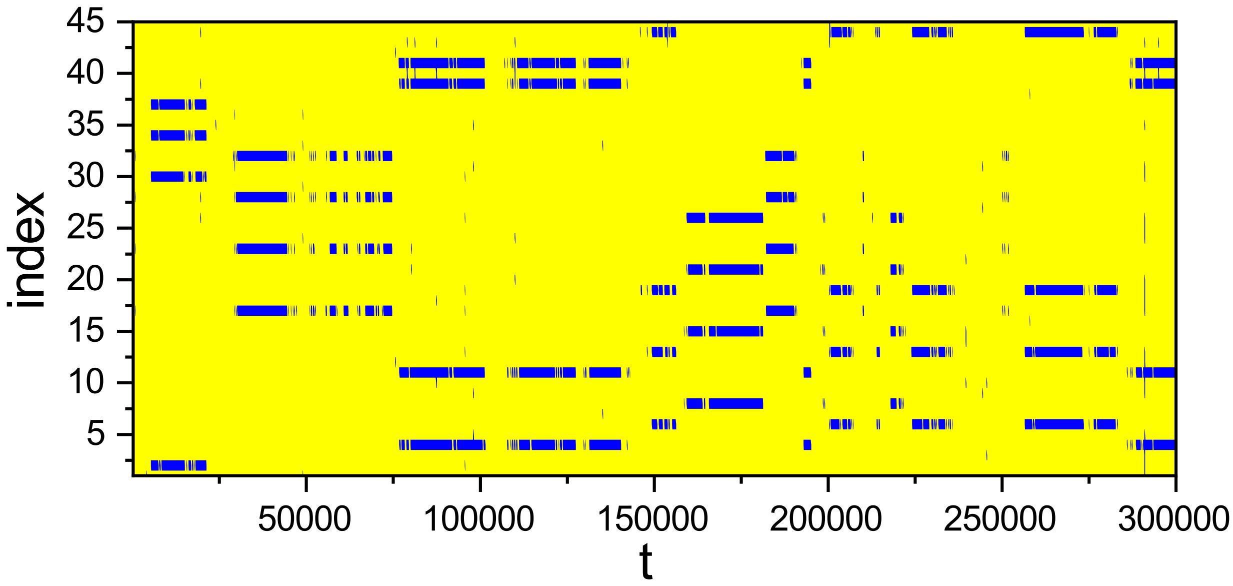

FIG. S8. *Transient cluster synchronization in coupled chaotic Rössler oscillators.* Shown is the time evolution of the synchronization relationship for coupling strength $\varepsilon = 0.046$. The network structure is identical to that in Fig. 1(a) in the main text.

X. INVERSE CUMULATIVE DISTRIBUTION OF TRANSIENT LIFETIME

To further elucidate the power-law distribution of the transient lifetime, we calculate the inverse cumulative distribution [1]. Specifically, let $f(x)$ be the probability distribution of a random variable x. The inverse cumulative distribution determines the possibility for finding an event larger than certain value of x: $P(x) = \int_x^\infty f(x)dx$. If $f(x)$ follows an algebraic scaling, we have $f(x) \sim x^\gamma$, so the inverse cumulative distribution follows an algebraic scaling: $P(x) \sim x^\Gamma$, with $\Gamma = \gamma + 1$. Figure S9 shows the cumulative distribution calculated from the same time series of transient lifetime as that in Fig. 3 in the main text. In the interval $T_{CS} \in (0, 10^2)$ (the same interval used in the main text for fitting the distribution), the distribution can be fitted by an algebraic scaling with $\Gamma \approx -0.58$. For comparison, Fig. S9 also includes the lifetime distribution shown in Fig. 3 in the main text. We have $\Gamma \approx \gamma + 1$. The thin tail in the inverse cumulative distribution is due to the limited data (a finite-size effect).

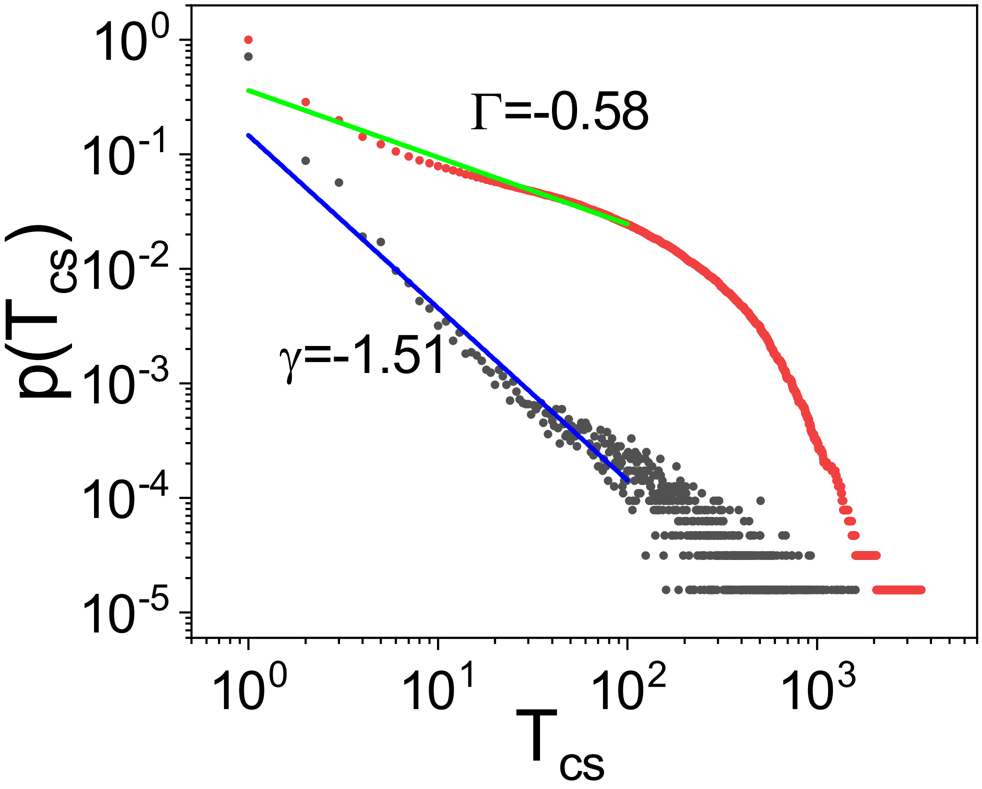

FIG. S9. *Inverse cumulative distribution of transient lifetime.* In the interval $T_{CS} \in (0, 10^2)$, the distribution can be fitted by an algebraic scaling: $p(T_{CS}) \sim T_{CS}^\Gamma$ with $\Gamma \approx -0.58$ (red dots and green line). To facilitate a comparison, the probability distribution in Fig. 3 of the the main text is also shown (black dots and blue line).

XI. VARIATION OF DEGREE OF SYNCHRONIZATION ABOUT ε_c^{CS}

For the food web network studied in the main text, in the region where transient cluster synchronization occurs, i.e, $\varepsilon \leq \varepsilon_c^{CS} \approx 0.4$, there is little change in the degree of global synchronization, as shown in Fig. S10, where the degree of synchronization is characterized the error defined as

$$\delta X = \sum_1^N [(x_i - \langle x \rangle)^2 + (y_i - \langle y \rangle)^2 + (z_i - \langle z \rangle)^2]^{1/2}/N,$$

where (x_i, y_i, z_i) is the state of the ith patch and $\langle x \rangle = \sum_i^N x_i$, $\langle y \rangle = \sum_i^N y_i$, and $\langle z \rangle = \sum_i^N z_i$ characterize the network averaged state. The behavior in in Fig. S10 is expected, as the impact of increasing the coupling strength is to extend the lifetime of the cluster synchronizations states, whereas the switchings between the different cluster synchronization states do not affect the degree of global synchronization since these states have the same δX value.

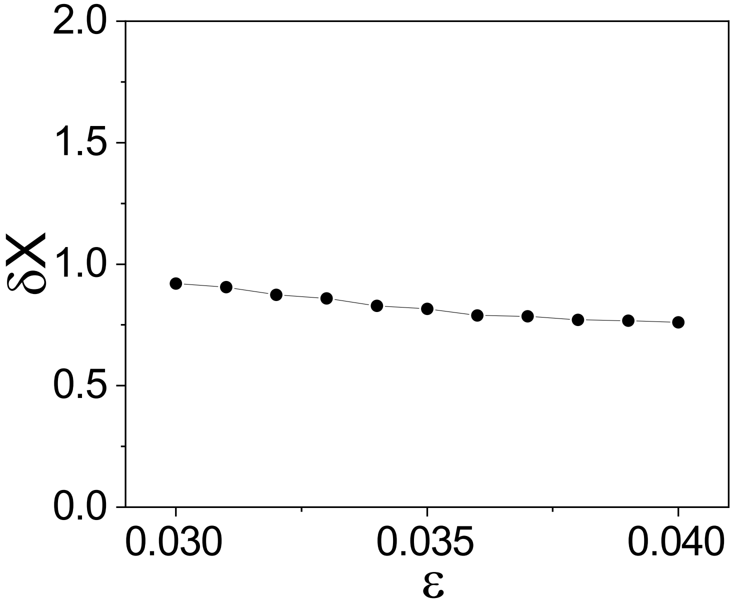

FIG. S10. *Behavior of the global synchronization error in the food web network.* Shown is δX versus ε about the critical coupling $\varepsilon_c^{CS} \approx 0.04$. In the region where transient cluster synchronization arises, i.e., $\varepsilon \in (0.035, \varepsilon_c^{CS})$, the value of δX is approximately constant. Each data point is the result of averaging over a time period of 10^4 cycles of oscillation.

XII. EFFECT OF COUPLING ON STATISTICAL PROPERTIES OF SYNCHRONIZATION MANIFOLD

Figure 7 in the main text demonstrates that, prior to the regime of transient cluster synchronization, the conditional Lyapunov exponent Λ fluctuates and crosses zero multiple times. The fluctuations are induced by the deformation of the synchronization manifold: they do not imply any new type of synchronization transition. To provide support, we show in Figs. S11 and S12 typical trajectories from patch 1 in the reduced network (Fig. 7 in the main text) for several values of the coupling strength in the fluctuating region. It can be seen that, at exactly the points where Λ becomes negative, the attractor is deformed from that of the isolated oscillator. On the contrary, in the range where transient cluster synchronization arises, i.e., $\varepsilon \in (0.035, 0.04)$, the statistical properties of the synchronization manifold are characteristically similar to those of the isolated attractor, leading to a smooth decrease in Λ with the increase of ε.

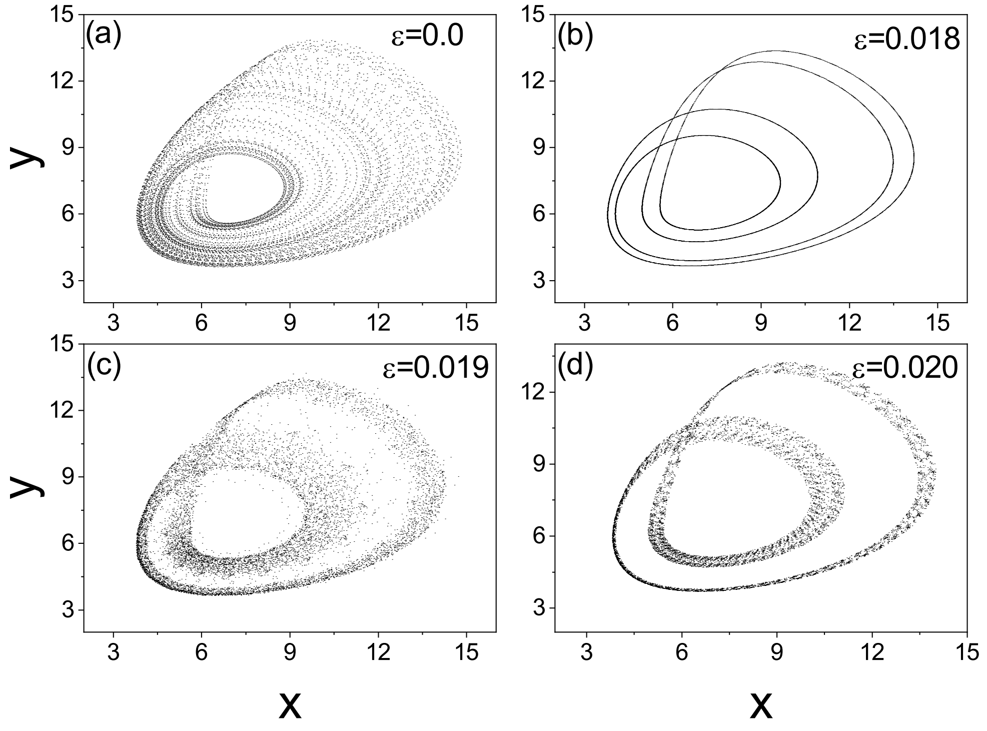

FIG. S11. *Typical trajectories from the parameter region where there are fluctuations of the conditional Lyapunov exponent*. For the chaotic food web network, in the coupling parameter range $\varepsilon \in (0.018, 0.03)$, fluctuations of the conditional Lyapunov exponent occur, due to the deformation of the synchronization manifold. Shown are typical trajectories from the first patch in the reduced network [Fig. 7(a) in the main text] for different values of the coupling strength: (a) $\varepsilon = 0$, (b) $\varepsilon = 0.018$, (c) $\varepsilon = 0.019$, and (d) $\varepsilon = 0.02$.

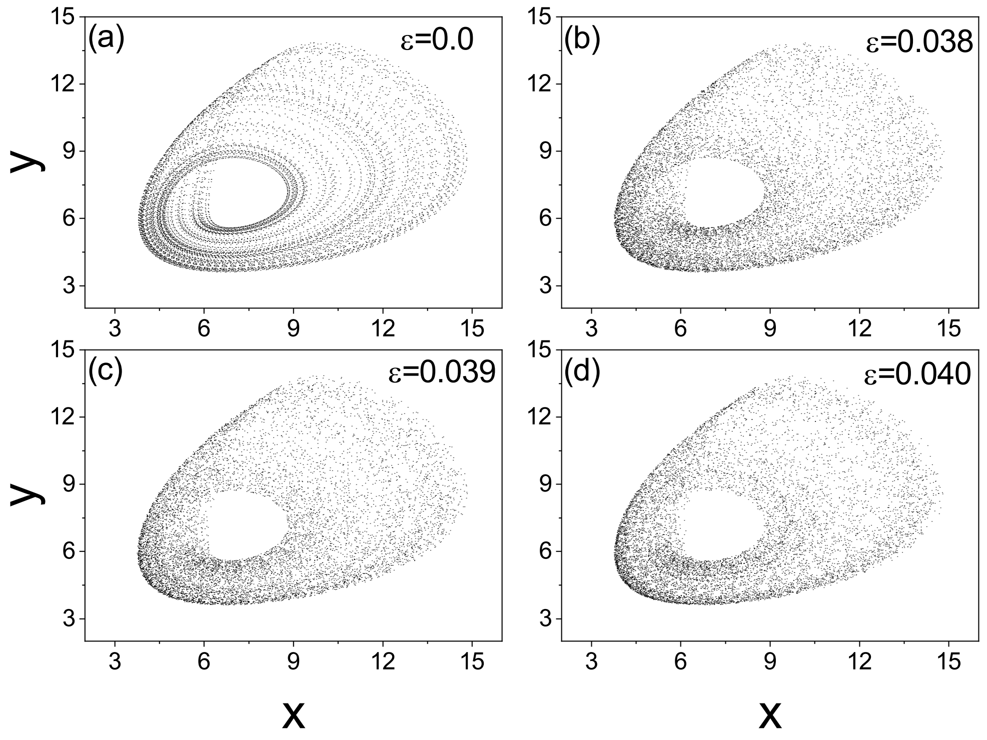

FIG. S12. *Typical trajectories from the parameter region where there is transient cluster synchronization.* For the chaotic food web network, in the parameter interval $\varepsilon \in (0.035, 0.04)$ where transient cluster synchronization arises, the synchronization manifold remains statistically unchanged. Shown are typical trajectories from the first patch in the reduced network [Fig. 7(a) in the main text] for different values of the coupling strength: (a) $\varepsilon = 0$, (b) $\varepsilon = 0.038$, (c) $\varepsilon = 0.039$, and (d) $\varepsilon = 0.04$

XIII. EFFECT OF SYMMETRY PERTURBATIONS ON TRANSIENT BEHAVIORS

Transient cluster synchronization persists when the network symmetry is slightly broken. To provide supporting evidence of the robustness of the phenomenon of transient cluster synchronization against symmetry-breaking perturbations, we introduce random perturbations of magnitude 1% to the parameter b in the chaotic food web network so that there is a slight parameter mismatch. Figure S13(a) shows the result, where the cluster synchronization states are slightly smeared, but the intermittent behavior is still apparent. Similar results are obtained when random perturbations are applied to the network couplings, as shown in Fig. S13(b), where the magnitude of the perturbations is 5% of the original value of the coupling strength.

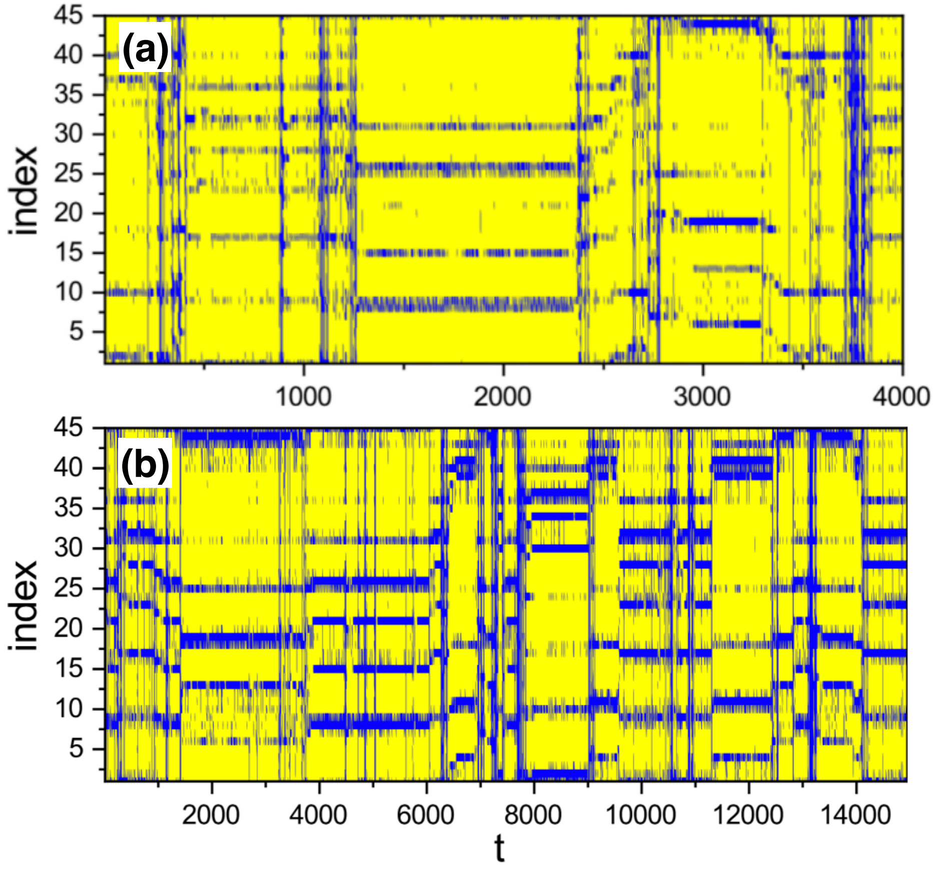

FIG. S13. *Effect of symmetry-breaking perturbations on transient cluster synchronization.* The network system is the same as the one in Figs. 1 and 2 in the main text. Shown are the time evolution of the dynamical variables of the system for $\varepsilon = 0.038$ with: (a) random perturbations of magnitude 1% in the parameter b, and (b) random perturbations of magnitude 5% in the coupling parameter.

[1] White, E. P., Enquist, B. J. & Green, J. L. On estimating the exponent of power-law frequency distributions. *Ecology* **89**, 905–912 (2008).
